# Young adults’ perspectives on chlamydia-related subfertility and a potential predictive subfertility test: A mixed-methods study in the Netherlands

**DOI:** 10.1371/journal.pone.0351874

**Published:** 2026-06-18

**Authors:** Bernice M. Hoenderboom, Zoïe W. Alexiou, Charlotte M. M. Peters, Karlijn Kampman, Colette van Bokhoven-Rombouts, Christian J. P. A. Hoebe, Birgit H. B. van Benthem

**Affiliations:** 1 Centre for Infectious Disease Control, National Institute for Public Health and the Environment, Bilthoven, The Netherlands; 2 Institute for Public Health Genomics, Genetica & Cell Biology, Maastricht University Faculty of Health Medicine and Life Sciences, Maastricht, The Netherlands; 3 Department of Social Medicine, Care and Public Health Research Institute, Maastricht University, Maastricht, The Netherlands; 4 Department Sexual Health, Infectious Diseases and Environmental Health, Living Lab Public Health Mosa, Public Health Service South Limburg, Heerlen, The Netherlands; 5 Public Health Service Twente, Enschede, The Netherlands; 6 Sexual Health Center, Public Health Service Gelderland-Zuid, Nijmegen, The Netherlands; 7 Department of Medical Microbiology, Infectious Diseases and Infection Prevention, National Chlamydia trachomatis Reference Laboratory, Care and Public Health Research Institute (CAPHRI), Maastricht University Medical Center (MUMC+), Maastricht, The Netherlands; GGD Amsterdam, NETHERLANDS, KINGDOM OF THE

## Abstract

**Background:**

*Chlamydia trachomatis* (chlamydia) infection control could be more effective if focused on morbidity- rather than prevalence reduction. A predictive test, identifying those at higher risk for chlamydia-subfertility, could be developed to enhance targeted chlamydia control. However, do young adults with a uterus (hereafter “young adults”) want to know their subfertility risk?

**Methods:**

A sequential mixed methods study was conducted among young adults in the Netherlands to explore perspectives on chlamydia, subfertility, and a potential subfertility test. Five focus groups with Sexual Health Center (SHC) visitors were held to identify potential benefits, barriers, and requirements; results were analysed thematically and used to inform a questionnaire. This questionnaire was distributed online via SHCs and social media. Descriptive statistics and modified Poisson regression were used to assess benefits and barriers of learning one's subfertility risk.

**Results:**

Nineteen young adults participated in the focus groups, and the resulting themes informed the questionnaire. These included perceived benefits (mental preparation, anticipation, reassurance after a “no increased risk” result), perceived barriers (mental burden of an “increased risk” result, need for blood sampling), and requirements (accuracy, accessibility, follow-up). The questionnaire was completed by 426 participants (median age 22, IQR 20–24). High perceived susceptibility was reported by 11% for chlamydia and 23% for chlamydia‑related subfertility; perceived severity was high for chlamydia and chlamydia-related subfertility, 75% and 88%, respectively. Willingness to use a risk test was 78%. Relief after a “no increased risk” result was expected by 89%, while an increased‑risk result was expected to enhance preparedness (86%) and elicit worry (83%). Additional considerations included motivation for safer sex, negative effects on relationships, and questioning the value of knowing.

**Conclusion:**

Young adults are concerned about chlamydia‑related subfertility, resulting in high willingness to use a risk test. The expected benefits and barriers should be carefully considered in test development.

## Introduction

*Chlamydia trachomatis* (chlamydia) is the most reported bacterial sexually transmitted infection (STI) worldwide, despite a range of control activities such as (opportunistic) screening [1–4]. An evolving understanding of chlamydia disease progression has led to an ongoing debate about the most effective and efficient methods of chlamydia control [1–3]. Approximately 5% of infected women develop complications such as pelvic inflammatory disease, ectopic pregnancy, or tubal factor infertility [5–7]. Large scale screening trials in the Netherlands and Australia did not demonstrate a cost-effective impact on reducing prevalence [8,9], and evidence for a population‑level reduction of screening in chlamydia complications is weak [2,8,10]. Because long-term complications are relatively rare, many asymptomatic individuals may be treated unnecessarily, contributing to antibiotic overuse [2]. Shifting the focus from prevalence reduction to morbidity reduction in the small proportion of women who develop complications may therefore be more effective [1–3].

To enable such a shift, it is necessary to identify which women have an increased risk of developing long-term complications. Several research groups in the USA [11] and EU [12] are actively investigating genetic markers that can determine an individual’s predisposition to chlamydia infection and its long-term complications [12–17]. The ultimate goal is to develop a prognostic risk tool [12] that integrates genetic information with other relevant risk factors, including immunological markers [18–20], health characteristics [21], infection characteristics [5] and demographic variables [17]. Estimating individual risk for complications may offer several benefits. Awareness of increased risk can reduce risk behavior, such as preventing re-infection, which increases complications risk [6,22]. Improved fertility knowledge decreases misconceptions and supports reproductive decision-making [23,24].

Ultimately, the value of a predictive infertility test depends not only on its diagnostic performance but also on its acceptability among the target population, namely young adults with a uterus. Test uptake depends on perceived disease risk and on perceived benefits and barriers of testing [25]. Therefore, during development of such a test, it is important to understand the specific needs, requirements, motivations and barriers of young adults to use such a test [26]. This study aimed to explore young adults’ perspectives on chlamydia, chlamydia-related subfertility and their anticipated needs, perceived benefits, and potential barriers to a predictive subfertility test.

## Materials and methods

### Study design

We performed a sequential mixed-methods study. Phase 1 included developing a questionnaire based on behavioral theory frameworks, expert opinions, focus groups and testing of the questionnaire. In phase 2 we used the developed questionnaire to identify perceptions on susceptibility and severity of chlamydia (complications) and to assess attitudes, barriers and benefits to a predictive test. In this study, ‘young adults’ refers to young adults with a uterus, regardless of their gender identity. Furthermore, we use the term “subfertility” rather than “infertility” because it more accurately reflects the clinical situation [27]; even in the presence of tubal damage, pregnancy remains possible [28].

### Phase 1: Development of the questionnaire

#### Frameworks and expert opinions.

**Fig 1:**
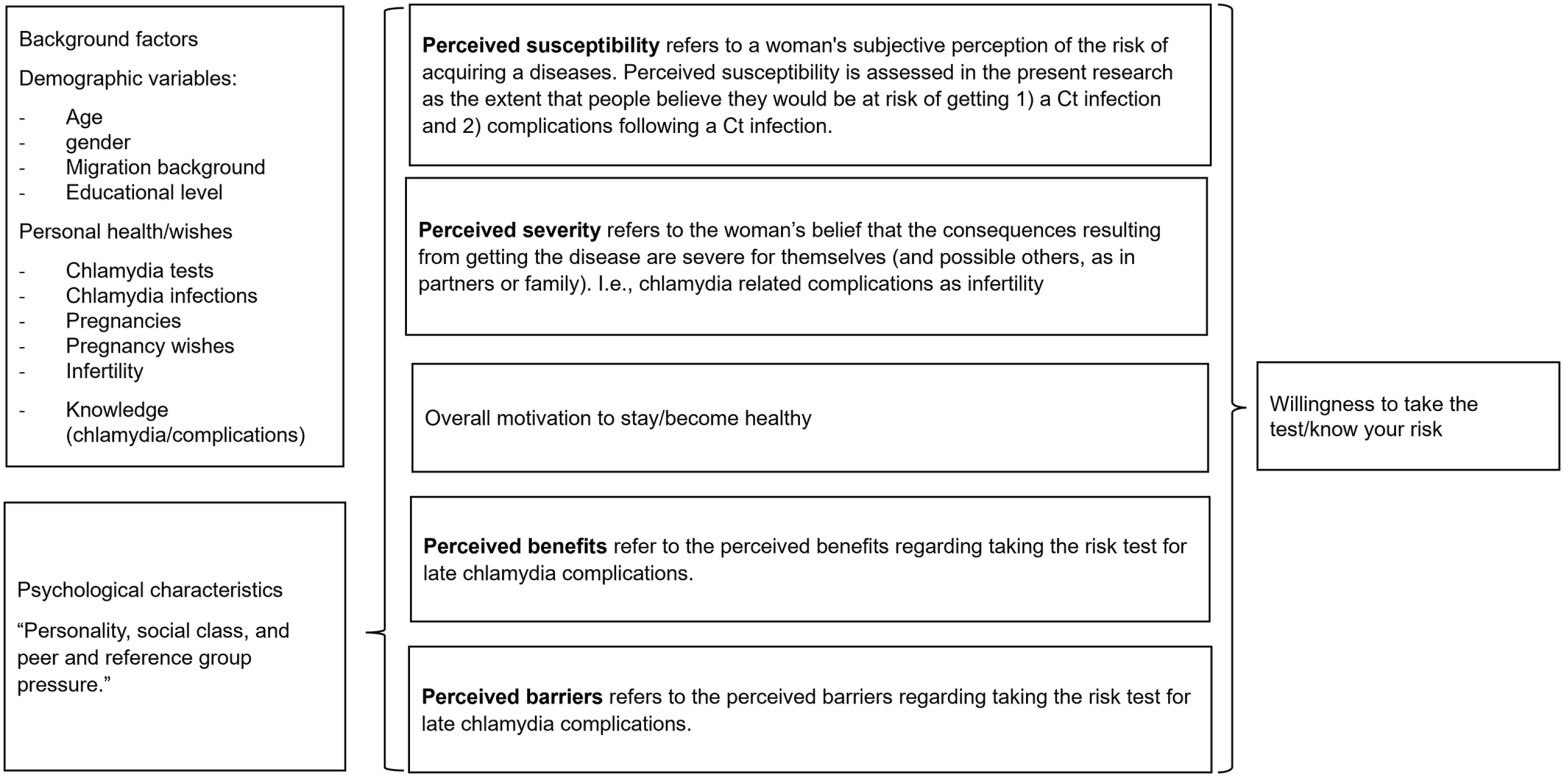
The Health Belief model including elements of the Reasoned action model adapted to the Chlamydia predictive subfertility test (Rosenstock, 1974, Zampetakis, 2021 & Fishbein, 2010).

The questionnaire was based on the Health Belief Model [25], supplemented by elements of the Reasoned Action Model [29], and adapted to the predictive chlamydia subfertility test (Fig 1). The questionnaire assessed background information, (including demographics, sexual behavior, STI (testing) history, prior chlamydia knowledge, and attitudes on chlamydia prevention and population screenings).

Risk perception was assessed through perceived susceptibility, i.e., perceived risk of acquiring a chlamydia infection and becoming sub-fertile following chlamydia, and perceived severity, i.e., the subjective assessment of the severity of the chlamydia infection and subfertility. Willingness to take the test and the perceived benefits and barriers were also assessed; these perceived benefits and barriers were partly informed by focus groups input. Information about the potential test was provided in the questionnaire (S1) in S1 File.

Questions on risk perception, health goals and impulsivity were based on a validated questionnaire from van Wees et al. [30]. Impulsivity, particularly the “negative urgency” facet from the UPPS-P scale [31], was included because it has been linked to chlamydia diagnoses [32] and sexual behavior linked to STIs [33,34]. Health-related goals, which influence decision-making and engagement in sexual behaviors linked to STIs were assessed using items from Den Daas et al. [35].

Health goals, impulsivity and risk perception were all measured using a 5-point Likert scale. The questionnaire items can be found in S1 File.

#### Focus groups to identify barriers and benefits.

As no previous studies have examined benefits and barriers related to an STI subfertility risk test, we performed exploratory qualitative research with the target population to identify barriers and benefits of such tests through focus groups (Table 1).

**Table 1:** Design of the focus group.

Study population	The focus groups were held with young adults who had recently undergone an STI test in the age of 18–25. The purposive sampling emphasized an equal distribution between young adults with a practical and theoretical education [36]. Young adults were informed and recruited by healthcare professionals during SHC consultations in various geographical areas in the Netherlands (Rotterdam, Maastricht, Nijmegen and Enschede).
Data collection	The topic guide for the focus groups was developed in accordance with the questionnaire using the health belief model (Fig 1). The main topics were: prior knowledge on STIs/chlamydia, risk perception of chlamydia (-complications), needs and desires of knowing your subfertility risk for context and understanding and focused on the barriers and benefits of a predictive test (topic list & program (S2): in S1 File and test information (S3) in S1 File). The focus groups were held physically at the SHC in a private room. Two to three female researchers aged 29–34 years old were present. One of them was leading the focus group. Focus groups lasted between 1–1.5 hour. Focus groups were held between June 6 – August 30 2023.
Data analysis	Focus groups were recorded by a voice recorder, transcribed verbatim and anonymized by the researchers who conducted the focus groups (BMH, ZWA). Analysis followed a thematic approach as described by Braun and Clarke (2006) [37] and was done in Atlas.ti 24. Our thematic analysis process involved a hybrid of both inductive and deductive coding. The coding framework was primarily based on the interview guide, with emergent codes being added as identified during the analysis. Coding was done by (ZWA) and reviewed by one of two (experienced) researchers (BMH, CMMP) and discussed until an inter-coder agreement was reached. After coding, themes were identified by searching for patterns and sorting codes into different themes (BMH), guided by the adapted HBM. The themes were iteratively reviewed, discussed, refined and defined.

Findings from the focus groups were used to construct predefined benefits and barriers in the questionnaire, although participants first responded to open‑ended questions to elicit their own perspectives before viewing the predefined items. The focus groups were also used to improve our understanding of young adults’ conceptualization of the topic, which guided the formulation and wording of the questionnaire items.

The development of the questionnaire, including the incorporation of theoretical models and elements from literature, was iteratively discussed and reviewed by an advisory committee consisting of behavioral scientists, medical doctors, STI experts, epidemiologists and a representative of Freya, a Dutch patient organization representing people with fertility problems.

#### Testing of the questionnaire.

To ensure the questions were interpreted as intended, the questionnaire was tested through two rounds of cognitive interviews with four young adults with practical/vocational education [38]. The think-aloud method and verbal probing techniques were used [38]. The final questionnaire was reviewed by professionals from the SHCs to ensure wording was consistent with their websites and triage questionnaires.

### Phase 2: Roll out of the questionnaire

#### Study population & collection.

The online questionnaire developed in phase 1 was aimed at young adults with a uterus aged 18–25 who had ever engaged in vaginal intercourse with cisgender male. Recruitment efforts included targeted social media advertisements on Facebook and Instagram, with adjustments made based on response rates. Additionally, clients of SHC were invited to participate through a survey link provided in the online triage system when making an appointment for sexual health consultation, text message appointment reminders, and via flyers and posters displayed at the SHC. In total, 15 of the in total 24 SHCs geographically spread across the Netherlands participated.

Participants who completed the questionnaire were offered a 5-euro gift card. Recruitment took place between April 1, 2024, to May 15, 2024.

### Data analysis

#### Statistical analysis.

Participants’ characteristics were stratified by chlamydia history (ever diagnosed yes/no) and compared using student’s T test, Mann-Whitney U-test, chi-squared tests or Fisher’s exact test.

Risk perception scores based on a 5 point Likert scales were divided into low, neutral and high categories to ensure sufficient numbers in each group. Continuous variables (average health goals score, average impulsivity score, average chlamydia knowledge score, average attitude towards chlamydia and subfertility prevention score and attitude towards population screening) were divided at the median in two categories in alignment with previous research [39]. Variables on wanting to know their subfertility risk and willingness to take the potential risk test were divided in “yes” (completely agree and agree) and “no” (completely disagree, disagree, not agreeing/not disagreeing).

Univariable and multivariable modified Poisson regression analyses (i.e., Poisson regression with a robust error variance) were conducted to identify factors associated with the willingness to take the potential risk test. The modified Poisson regression was used to avoid overestimation of the associations due to the high outcome probability [40]. All variables (Table 3) were included in the univariable analyses. The multivariable model was constructed using backward selection based on the Akaike Information Criterion (AIC), including variables significantly associated in univariable model (p < 0.05). Multicollinearity was accounted for using the Variance Inflation Factors (VIF) command using a threshold of >5 as an indicator for multicollinearity. Results were reported in relative risks (RR) and adjusted relative risks (aRR). Quantitative analyses were performed in STATA version 18.

#### *Open text analysis*.

In the questionnaire, young adults were asked to report in open text boxes potential benefits and barriers to the potential subfertility risk test. To analyse these, we used inductive content analysis (ICA) which is commonly used for text-based data [41]. First, in Excel the answers were read extensively and only narratives that included views regarding benefits or barriers to the risk test were considered relevant. Following the preparation, open coding was conducted. The codes were meant to represent the meaning of the answers [41]. One hundred random answers were coded blindly by BMH and ZWA and compared, whereafter BMH completed the coding. Within the codes we searched for patterns and themes and sorted the codes among the themes. The themes were iteratively reviewed, compared, discussed and refined [41]. Themes were named based on the information they contained.

### Ethics statement

At start of the focus group study information was reiterated. It was confirmed that participation was voluntary by at least two researchers and could be stopped at any time, and consent was implied through focus group involvement and recorded. Participants had sufficient time before the focus group to read the information letter and ask questions. This method of consent was approved by lawyers of the internal Privacy Team of the RIVM. In addition, this study has been reviewed by the Medical Ethical Research Committee Amsterdam University Medical Center, Amsterdam, the Netherlands, (no. 2023.0181) and deemed exempt from further IRB review under the Dutch Medical Research Involving Human Subjects Act (WMO). All participants provided informed consent prior to participation: orally for the focus groups and in writing for the questionnaire.

## Results

### Focus groups

A total of 19 young adults participated in five focus groups (range 2–5 participants). The median age of the young adults was 22 years (range 18–25). Nearly half of the young adults (47%) had a practical education background (see Table 2).

**Table 2:** Demographics of focus group with young adults (N = 19).

		N, (%) or range
Age in years	(median, range)	22 (18 - 25)
Location	Enschede	7 (36.8%)
	Rotterdam	4 (21.1%)
	Maastricht	5 (26.3%)
	Nijmegen	3 (15.8%)
Occupation	Studying	9 (47.4%)
	Working	6 (31.6%)
	Working & studying	2 (10.5%)
	Neither	2 (10.5%)
Education	Theoretical eduation	10 (52.6%)
	Practical education	9 (47.4%)

**Fig 2:**
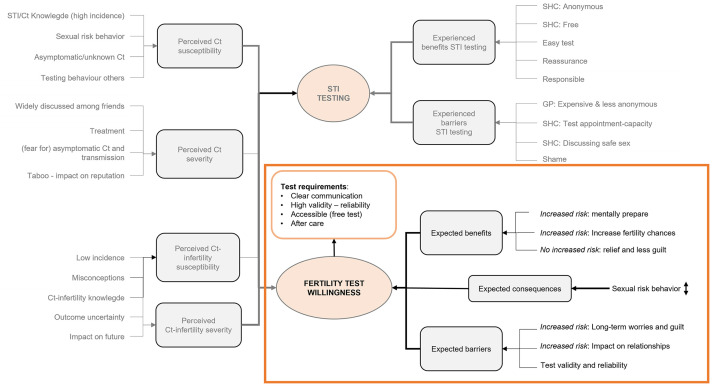
Illustration of the relation between perspectives and test willingness from focus group data. SHC = Sexual Health Clinic, GP = General Practicionar, Ct = Chlamydia trachomatis, STI = sexually transmitted infection. The results shown in the orange box were taken into account when developing the questionnaire. The factors listed after the italicized items indicate the benefits associated with the corresponding italicized test result.

Below, we discuss “chlamydia-subfertility testing attitude and benefits”, “requirements of the subfertility test”, “chlamydia-subfertility testing barriers”, and “expected consequences of the subfertility test”. Conceptual focus group findings (S4) are available in S1 File. Fig 2 illustrates the relation between perspectives and test willingness from focus group data.

#### Chlamydia-subfertility testing attitude and benefits.

The potential subfertility test was perceived as a positive contribution to sexual health(care).

- Mental preparation and timing

Many young adults believed it is better to know of an increased subfertility risk following chlamydia at a young age compared to when you start having children. In contrast, several young adults mentioned that the timing of the test might be better when they are thinking about children or when they want to get pregnant. Main benefits to knowing their subfertility risk were: to be able to prepare mentally, to have less uncertainty and to get reassurance.

*“I think that women are keen on it [the test] because it [chlamydia] can give you insecurity and a bad feeling. If you can take away even a little bit of the insecurity, I think that is nice, especially for young girls” FG 5, P5*

- Anticipating on results

Some young adults mentioned that they can take action if they would know they have an increased risk for subfertility. Actions that were mentioned were: starting earlier trying to get pregnant, freezing your eggs, IVF treatment, exploring medical options, saving money for fertility treatments, exploring options with your partner and discussing adoption. Another benefit mentioned was having the possibility to stop using contraceptives. Some women incorrectly believed that, in that case of subfertility, contraceptives might not be necessary anymore.

*“I think it is good to know now. Rather now than when I start trying to get pregnant and it doesn’t work. I think I find it very difficult to know, but if you think about the long term, you can visit a doctor in advance that might help you or find another way to get pregnant or explore different options.” FG 3, P3*

- Positive feelings

Knowing you don’t have an increased risk could result into feelings of relief, less guilt and might give peace of mind. On the other hand, young adults expected that it would not change much but it would be rather “nice to know”.

#### Chlamydia-subfertility testing barriers.

- Mental burden

Expected barriers to knowing you have an increased risk of subfertility included prolonged feelings of worry, unhappiness, or guilt. Some young adults feared feeling less feminine or believed it might have negative impacts on future relationships, such as uncertainty whether or when they should inform a partner.

*“.. but I would also, my very first reaction was oh I don’t know if I would want to know if you can’t change anything about it, because you might be very unhappy for a very long time like I might not have children and you can’t change it.“ (FG 2, P1)*

Additionally, the test might provide false hope since subfertility can be caused by factors other than chlamydia. Furthermore, receiving only an indication of a possible higher risk might add to the existing uncertainty rather than providing reassurance.

- Need for blood drawing

The test might require drawing blood, which is expected to be a barrier for young adults who are afraid of needles.

#### Requirements of the subfertility test.

- Accuracy

High accuracy was deemed crucial. Most young adults indicated they would only consider the test if it had a maximum error rate of 1 in 100, to prevent increased insecurity. Some young adults would accept a lower test accuracy, acknowledging that the test would only give an indication and not a definitive outcome. For some, the concept of test accuracy was too complicated to comprehend.

- Accessibility & communication

Accessibility was believed to be essential, with many young adults saying that the test should be free or at least affordable. Clear communication about the test would be essential. This includes information on how the test works, what is done with the data, the interpretation of the results, and potential follow-up options.

*“It depends how you frame it. […] if you very firmly say ok the test is correct 8 out of 10 times. But yes, than still I would question if the test is correct and I think that, you have to inform and explain exactly what you mean and, but 8 out of 10 is still a bit vague actually” FG 4, R4*

Finally, some form of after care is important and should be available.

#### Expected consequences of the subfertility test– risk behavior.

Young adults foresee that knowing they have an increased risk for subfertility might lead to practicing safer sex or undergoing more regular STI testing to prevent further complications. However, others might become less careful about preventing pregnancy, assuming they are less fertile. Similarly, knowing that they do not have an increased risk could motivate some to use condoms more consistently to keep the risk for a chlamydia infection low, while others might feel that safe sex is less relevant. Regardless of the test results it is believed that the test itself can raise awareness and promote safer sexual practices.

*“I think I would, if I found out I had an increased risk because of chlamydia, I would be more careful with unsafe sex than if I learned I didn’t have an increased risk. So in that case, getting a result that you’re not at increased risk might even make people think less about unsafe sex” F2, P1*

### Questionnaire

In total, 426 persons participated in the online survey, of which 389 (91.3%) were recruited via social media. Most participants identified as female (n = 416, 97.7%) and the median age was 22 (20–24). The majority was born in the Netherlands (n = 361, 84.7%) and was theoretically educated (n = 344, 80.8%). One third of the participants ever had a chlamydia infection (n = 138, 32.4%), see Table 3.

**Table 3:** Characteristics of the survey-study population by chlamydia status.

		Overall n (%)	Chlamydia-negative n (%)	Chlamydia-positive n (%)	p-value
		426 (100%)	288 (67.6%)	138 (32.4%)	
Gender					0.095*
Female		416 (97.7)	278 (96.5)	138 (100.0)	
Non-binary		6 (1.4)	6 (2.1)	0 (0.0)	
Male		4 (0.9)	4 (1.4)	0 (0.0)	
Age (years) median (IQR)		22 (20-24)	21 (20-23)	23 (21-24)	<0.001^@^
Migration background					0.350^%^
No migration background		361 (84.7)	247 (85.8)	114 (82.6)	
Migrant		19 (4.5)	14 (4.9)	5 (3.6)	
Child of migrant		46 (10.8)	27 (9.4)	19 (13.8)	
Educational level					0.882^%^
Practical		82 (19.3)	56 (19.4)	26 (18.8)	
Theoretical		344 (80.8)	232 (80.6)	112 (81.2)	
Age at sexual debut mean (SD)		16.9 (1.9)	17.1 (1.9)	16.5 (1.8)	0.001^$^
Number sex partners past six months					<0.001^%^
0-1		202 (47.4)	154 (53.5)	48 (34.8)	
2-3		121 (28.4)	79 (27.4)	42 (30.4)	
> 3		103 (24.2)	55 (19.1)	48 (34.8)	
Most recent partner type					0.001*
One-time partner		77 (18.1)	45 (15.6)	32 (23.2)	
Casual partner		147 (34.5)	87 (30.2)	60 (43.5)	
New steady partner		46 (10.8)	32 (11.1)	14 (10.1)	
Steady partner		154 (36.2)	122 (42.4)	32 (23.2)	
Sex worker		2 (0.5)	2 (0.7)	0 (0.0)	
Condom use at last sex act					<0.001*
Always		22 (5.2)	21 (7.3)	1 (0.7)	
Usually yes		115 (27.0)	90 (31.3)	25 (18.1)	
Sometimes		105 (24.7)	59 (20.5)	46 (33.3)	
Usually no		156 (36.6)	97 (33.7)	59 (42.8)	
Never		28 (6.6)	21 (7.3)	7 (5.1)	
STI test history					<0.001*
Never		115 (27.0)	114 (39.6)	1 (0.7)	
Yes, in the past six months		138 (32.4)	65 (22.6)	73 (52.9)	
Yes, over six months ago		173 (40.6)	109 (37.9)	64 (46.4)	
Gonorrhoea history					0.007^%^
No		397 (93.2)	275 (95.5)	122 (88.4)	
Yes		29 (6.8)	13 (4.5)	16 (11.6)	
Wish for children					0.352^%^
Yes, now or later		322 (75.6)	212 (73.6)	110 (79.7)	
No/not anymore		56 (13.2)	42 (14.6)	14 (10.1)	
I don’t know		48 (11.3)	34 (11.8)	14 (10.1)	
Ever pregnant					0.085^%^
No/I don’t know		406 (95.3)	278 (96.5)	128 (92.8)	
Yes		20 (4.7)	10 (3.5)	10 (7.3)	
Health goals^#^					0.030^%^
Low (score ≤ 3.83)		163 (38.3)	100 (34.7)	63 (45.7)	
High (score >3.83)		263 (61.7)	188 (65.3)	75 (54.4)	
Impulsivity^&^					0.174^%^
Low (score ≤2.5)		224 (52.6)	158 (54.7)	66 (47.8)	
High (score >2.5)		202 (47.4)	130 (45.1)	72 (52.2)	
Perceived chlamydia susceptibility					<0.001^%^
Low		307 (72.1)	225 (78.1)	82 (59.4)	
Medium		73 (17.1)	43 (14.9)	30 (21.7)	
High		46 (10.8)	20 (6.9)	26 (18.8)	
Perceived chlamydia severity					<0.001^%^
Not severe (at all)		19 (4.5)	7 (2.4)	12 (8.7)	
Neutral		86 (20.2)	49 (17.0)	37 (26.8)	
(very) severe		321 (75.4)	232 (80.6)	89 (64.5)	
Perceived chlamydia-subfertility susceptibility					0.026^%^
Low		91 (21.4)	51 (17.7)	40 (29.0)	
Medium		236 (55.4)	165 (57.3)	71 (51.5)	
High		99 (23.2)	72 (25.0)	27 (19.6)	
Perceived chlamydia-subfertility severity					0.006*
Not severe (at all)		23 (5.4)	22 (7.6)	1 (0.7)	
Neutral		30 (7.0)	20 (6.9)	10 (7.3)	
(very) severe		373 (87.6)	246 (85.2)	127 (92.0)	

SD = standard deviation, IQR = interquartile range, # Health goals average score divided in high and low/med based on median split. & Impulsivity average score divided in high and low/med based on median split. *Fisher exact test used, ^@^Mann-Whitney test used, ^%^Chi-squared test used, ^$^Student’s t-test used.

#### Perceived susceptibility and severity.

Of all young adults, 382 (92.7%) knew that a chlamydia infection can be asymptomatic and 364 (88.4%) knew that chlamydia might cause subfertility. In contrast, 67 (16.2%) knew that chlamydia can resolve without treatment. Chlamydia *susceptibility* was perceived as high by 46 (10.8%, CI 8.0%−14.1%). *Susceptibility* to subfertility following chlamydia was perceived as high by 99 (23.2%, CI 19.3%−27.5%). S*everity* of chlamydia was perceived high by 321 young adults (75.4%, CI 71.0%−79.4%) and 373 young adults perceived a high severity of chlamydia subfertility (87.6%, CI 84.0%−90.5%).

#### Open-ended texts – test benefits and barriers.

Almost all reported benefits (answered by n = 401, 94.1%) and barriers (answered by n = 389, 91.3%) to knowing an individual's subfertility risk were framed in terms of having *an increased risk* for subfertility. Only two responses (0.3%) considered the scenario of *no increased risk* for subfertility.

*Benefits* to knowing your risk

Identified benefits presented in themes were: awareness, the possibility to anticipate, motivator for safe sex, less insecurity and quitting contraceptive use for knowing you have an increased risk. And “relief” if you don’t have an increased risk.

*Awareness* of one’s subfertility risk was seen as a benefit in two ways. Respondents wanted to be aware of their own health and physical condition, and know what they could expect. The test could also raise awareness of chlamydia and its consequences.

*The possibility to anticipate* was reported by many. First, being prepared and knowing what to expect was mentioned. It was also seen as useful to prepare mentally, to adjust your expectations and make better life choices. Some thought it would be easier to learn about your fertility chance before attempting to get pregnant. Furthermore, it was often mentioned that one would try to increase their fertility chances, by starting getting pregnant earlier, freezing eggs and preventing new STI’s. Additionally, the ability to involve and discuss this with a (future) partner, to prepare financially and to consider alternatives were mentioned.

A large benefit reported was feeling *less insecure*. This was reported in two ways. First, knowing the risk, instead of being in doubt, gives clarity. Second, knowing you have an increased risk for subfertility decreases the fear of unwanted pregnancies.

A reported benefit was the test as a *motivator for safe sex*. It was mentioned that respondents would use condoms more often, prevent getting chlamydia (again), or test partner(s) for STI’s. However, the opposite was also reported by some: The test might result in lesser need for safe sex. This is related to the theme *quitting contraceptive use*. Several respondents reported that they might consider stopping to take (hormonal) contraceptives, thinking it is unnecessary as they “can’t get pregnant anymore”. In respondents without a wish for having children the test result of being susceptible to becoming subfertile might be seen as positive.

*Barriers* to knowing your risk

Identified barriers to knowing one’s individual subfertility risk presented in themes were: mental burden, impact on a new relationship, and questioning the value of knowing.

The main reported barrier for knowing was the *mental burden*. Many respondents expect to get stressed learning about the risk of reduced subfertility and expect to carry this stress for a long time. The stress itself could reportedly lead to even lesser chances of getting pregnant. Furthermore, self-blame and feelings of guilt were often mentioned, in addition to prolonged feelings of sorrow and fear. A respondent mentioned to possibly losing hope or giving up.

The result might also have an negative *impact on (new) relationships*. It was reported that this could make it more difficult to find a new partner or to get into a relationship. Some worry about being blamed by their partner, having to have a difficult conversation about it or that it might change the relationship.

Many respondents *question the value of knowing* one’s individual risk. Many believe that receiving an increased risk result could cause unnecessary stress, as it is not an definitive result. The information was suggested to be useless, because a decreased risk does not mean that you cannot get pregnant and furthermore you cannot act upon the result. Quotes corresponding to the themes can be found in Table 4.

**Table 4:** Quotes from questionnaire belonging to the potential subfertility tests benefits and barriers.

Themes		Quotes
**Benefits for knowing your risk**		
Awareness		*“More knowledge about my own body”.*
The possibility to anticipate		*“You are prepared that it [getting pregnant] might take longer and you might adjust your lifestyle a bit.”* “*I know that my partner wants children and if I’m infertile I don’t want to stop him from having children. He will need to find a different partner for his own happiness and child wish”.* *“I can create a more realistic image of the future, I will know what the cause might be if it [getting pregnant] doesn’t succeed, and I can think about other options for having kids and getting used to the idea.”*
Less insecurity		*“Less likely to have an accident if the condom breaks. I never want to have kids”.*
Motivator for safe sex		*“.. furthermore, off course prevent getting chlamydia, so use condoms more often and let the partners get tested”.*
Quitting contraceptive use		*“If I can’t get pregnant anymore, I don’t have to use an IUD. That would be very chill. Besides it would be interesting te know more about my body”.*
Relief		*“… in case of good news, then you know that and you are at ease”.*
**Barriers for knowing your risk**		
Mental burden		*“More stress, even before you are in the life stage for children”.* *“It might get even more in your head and then getting pregnant won’t work because of the stress”.* *“Very sad to hear and less hope that you might get pregnant”.*
Impact on (new) relationships		*“It can turn your life upside down and might cause difficulties in your relationship.”*
Question the value of knowing		*“It only gives you stress, there might be nothing wrong. If it is indeed more difficult, then we’ll figure it out later.”* *“A lot of stress and worries because of a probability calculation, that is not definitive or completely predictive.”* *“Ignorance is bliss”.*

#### Willingness to test for subfertility.

In total, 78.9% of young adults wanted to know their individual subfertility risk and 78.2% were willing to take the potential test. Predefined perceived benefits and barriers for the test are listed in Table 5. Almost 90% young adults would feel relieved in case of not having an increased risk and 85.5% expect to be able to better prepare for the future in case of an increased risk for subfertility.

**Table 5:** Perceived benefits and barriers for receiving a test results either “no increased subfertility risk” or “increased subfertility risk” following Ct.

Perceived benefits & barriers	
Test result: No increased risk for subfertility following Ct	Test result: Increased risk for subfertility following Ct
• 89.7% expect to feel relieved • 84.0% expect to feel less worried • 21.6% expect that this doesn’t change anything • 10.6% expect to use condoms less often	• 85.5% expect to be able to better prepare for the future • 82.6% expect to worry a lot • 62.9% expect to use condoms more often • 12.9% expect that this result doesn’t change anything

Ct = Chlamydia trachomatis.

Factors associated with a willingness to take the test were perceiving “being prepared” as a benefit (aRR 1.9, 95%CI 1.4–2.5), not knowing if they want to have children (aRR 1.3, 95%CI 1.1–1.4), having a more positive attitude towards subfertility prevention (aRR 1.3, 95%CI 1.1–1.4) and having ≥3 partners in the past six months (aRR 0.9, 95%CI 0.8–1.0), Table 6, and Tabel (S5) in S1 File.

**Table 6:** Factors associated with willingness to take the potential predictive subfertility test.

		Test-intention	Relative risk			Adjusted Relative risk			
		n/N (%)	RR	95%CI	P value	aIRR	95%CI	P value	
Age – tertiles									
18-21		156/199 (78.4)	1			1			
22-23		83/107 (77.6)	0.95	0.54-1.68	0.868	1.01	0.90-1.13	0.914	
24-25		94/120 (78.3)	1.00	0.57-1.73	0.990	1.01	0.91-1.12	0.852	
No. sex partners past six months									
0-1		170/202 (84.2)	1			1			
2-3		89/121 (73.6)	0.87	0.77-0.99	0.031	0.92	0.83-1.03	0.146	
> 3		74/103 (71.8)	0.85	0.75-0.98	0.022	**0.88**	**0.77-1.00**	**0.044**	
Child wish									
Yes, now or later		258/322 (80.1)	1			1			
No/not anymore		32/56 (57.1)	0.71	0.56-0.90	0.005	0.95	0.75-1.21	0.694	
I don’t know		43/48 (89.6)	1.12	1.00-1.25	0.049	**1.27**	**1.13-1.44**	**0.009**	
Perceived Severity subfertility									
Low		13/23 (56.5)	1						
Neutral		15/30 (50.0)	0.88	0.53-1.47	0.636				
High		305/373 (81.8)	1.45	1.01-2.08	0.046				
Attitude prevention subfertility									
Low		150/217 (69.1)	1			1			
High		183/209 (87.6)	1.27	1.14-1.40	0.000	**1.25**	**1.11-1.41**	**<0.001**	
Attitude population screening									
Low		110/168 (65.5)	1						
High		223/258 (86.4)	1.32	1.17-1.49	0.000				
No risk – less worries									
Disagree		41/68 (60.3)	1						
Agree		292/358 (81.6)	1.35	1.11-1.65	0.003				
No risk – feel relieved									
Disagree		23/44 (52.3)	1						
Agree		310/382 (81.2)	1.55	1.17-2.07	0.003				
No risk – no change									
Disagree		275/334 (82.3)	1						
Agree		58/92 (63.0)	0.77	0.65-0.90	0.001				
Risk – no change									
Disagree		303/371 (81.7)	1						
Agree		30/55 (54.6)	0.67	0.52-0.85	0.001				
Risk – more condom use									
Disagree		114/158 (72.2)	1						
Agree		219/268 (81.7)	1.13	1.01-1.27	0.030				
Risk – more worries									
Disagree		42/74 (56.8)	1						
Agree		291/352 (82.7)	1.13	1.01-1.27	0.030				
Risk – better prepared									
Disagree		26/62 (41.9)	1			1			
Agree		307/364 (84.3)	2.01	1.50-2.71	<0.001	**1.88**	**1.40-2.52**	**<0.001**	

RR = Relative risk. aRR = Adjusted relative risk. CI = Confidence Interval.

## Discussion

In this sequential mixed-method study we provided insight in young adults’ perspectives on *chlamydia trachomatis*, related subfertility, and expected needs, benefits and barriers to a predictive subfertility test. Chlamydia susceptibility was perceived high by 11% of participants, whereas more participants (23%) perceived susceptibility to chlamydia subfertility as high. The majority considered both chlamydia and chlamydia subfertility as serious conditions (75% and 88%, respectively). Willingness to test was high at 78% and was associated with feeling better prepared, a positive attitude towards prevention, uncertainty regarding future childbearing, and having had fewer sexual partners. Reported benefits of knowing one’s risk included feeling better mentally prepared, anticipating behavioral changes, such as increased condom use, and experiencing reduced uncertainty. However, participants also identified several barriers, including stress, feelings of guilt, potential negative impacts on relationships, and high demands regarding the accuracy of a predictive test.

Perceived susceptibility to chlamydia is slightly higher in our study compared to another Dutch study. They found that 5% of young adults perceive themselves as very likely to acquire chlamydia compared to 11% in our study. The difference may be explained by the difference in the study populations: our participants were older and had a higher STI risk profile (SHC population, thus eligible for an STI test vs the general population) [42]. Perceived severity of chlamydia was comparable (77% versus 75%) [42]. The fear of subfertility following chlamydia in women is a known issue and has been previously described [43–45]. Up to 60% of women express nervousness about infertility following a chlamydia diagnosis [46].

We found a high willingness to knowing one’s subfertility risk (provided that the test has a high accuracy). In a large qualitative study in Sweden, in which they determined pros and cons of fertility awareness, youngsters as opposed to older people were interested in fertility counselling and check-up [47]. In contrast to our study, most participants in the Swedish study were uncertain if they wanted to be aware of their fertility status before trying to conceive. This difference could potentially be explained by the fact that, in our study, young adults could already have an underlying reason that could have impacted their fertility, namely (the fear of) chlamydia. The identified pros and cons aligned closely with the benefits and barriers identified in our study on the chlamydia subfertility test: knowing the status, you could anticipate and/or feel relieved, but it might also cause (unnecessary) distress and negatively impacting conceiving. Interestingly, a similar benefit to knowing was reconsidering the need for contraceptives and a reduced fear of unintended pregnancies [47].

The need for clear communication about chlamydia and subfertility was an important result in this study. Solving the misconception that chlamydia can only be resolved with antibiotics might help young adults to reduce anxiety. A qualitative study among English students also emphasized this specific need for knowledge about the process of how STIs cause infertility [48]. Furthermore, some young adults incorrectly believed that, in case of having an increased risk of subfertility, contraceptives would no longer be necessary. Although this line of reasoning is logically consistent, an elevated risk does not imply that one will inevitably become infertile. Moreover, even when fallopian tubes are scarred, the probability of achieving pregnancy is not null [49]. However, receiving more information on fertility in general and on one's individual fertility could also increase anxiety. In a large RCT in Japan among reproductive aged men and women, researchers found that with gaining more knowledge on (in-)fertility, the proportion of people that felt anxiety doubled [50].

### Implications

Many young adults were willing to take the potential subfertility risk test to be able to prepare, and reduce uncertainty. However, it is uncertain whether this test can deliver on these benefits, as it offers no treatment options, at most risk-reduction measures (e.g., preventing re-infection). Furthermore, the test specifically targets chlamydia-related subfertility rather than addressing overall subfertility, which could create a false sense of security. The high accuracy requirements young adults have are most likely difficult to obtain, and it is questionable whether young adults are able to properly evaluate the impact for themselves after receiving results of such a test. Expected barriers mentioned were prolonged feelings of stress, self-blame, fear and sorrow. The test might lead to a nocebo effect (“adverse effects produced by expectations”) [51,52]. And who decides if benefits outweigh barriers? In a recently published report from the Dutch council for Health and Society, a critical consideration was given about the diagnosis expansion. Benefits of health screening/checks are often overestimated, e.g., attainable lifestyle changes. Conversely, people might be wrongly validated in their unhealthy habits. Furthermore, overdiagnosis might increase the already overburdened health care system [52]. Instead of offering predictive tools to address young adults’ concerns, tailored information and support in coping with unavoidable life risks may help [53]. Resilience mitigates negative effects of stress, it might be more effective to strengthening resilience rather than attempting to take away the uncertainty [54].

Our study had several strengths. First, the sequential mixed-methods design, starting with qualitative focus groups, enabled a clearer understanding of a complex topic [55]. The focus groups offered important insights into how young adults interpreted the questions and information about the test. These insights contributed to the creation of a well-informed questionnaire and supported the interpretation of its results [56]. Some limitations need to be considered. First, fully grasping the concept of a potential subfertility test might be difficult for young adults. This was less of a concern during the focus groups because of the possibility to explain in more detail and respond to what was said. In the questionnaire, although the vast majority of the answers were plausible, some open-ended answers showed that young adults believed the test would give a definitive result, such as being fully infertile and no longer needing contraceptives. Second, it is possible that young adults interested in chlamydia who already have concerns about chlamydia and/or subfertility were more willing to participate in the focus groups about this topic, potentially limiting the generalizability of the results. This applies less for the questionnaire since the recruitment was merged with another STI-related study, therefore the announcement was less fertility focused. However, questionnaire respondents mainly had theoretical education and were predominantly without a migration background. Conversely, in the focus groups theoretical and practical education was evenly represented. Lastly, we aimed to recruit eight participants per focus group, but the actual attendance ranged from 2–5 participants. The session with only two participants resembled more of a group interview than a traditional focus group. The smaller sample size may have prevented us from identifying all potential benefits and barriers of the test during the focus groups, meaning some factors may have been missed when constructing the questionnaire. To address this, participants were first asked to list benefits and barriers in open ended questions before being shown the focus group derived items. Reassuringly, the open text responses largely echoed the focus group findings confirming and validating findings [57].

## Conclusion

Young adults express a desire for clarity and control regarding chlamydia subfertility. Therefore, the willingness for a predictive subfertility test is high, but the benefits of such a test might be questionable and barriers may outweigh them. Clear communication about chlamydia, subfertility and possible treatments and increasing young adults’ resilience is called for to reduce misconceptions and support young adults’ in making their own sexual and reproductive choices.

## Supporting information

S1 File Supplementary materials including: questionnaire on the predictive subfertility test (S1), topic list and focus group program (S2), PowerPoint presentation on chlamydia subfertility and the potential predictive test (S3), focus group results (S4), and table of factors associated with willingness to use the predictive test (S5). (DOCX)

## References

[1] Dukers-Muijrers NHTM, Evers YJ, Hoebe CJPA, Wolffs PFG, de Vries HJC, Hoenderboom B, et al. Controversies and evidence on Chlamydia testing and treatment in asymptomatic women and men who have sex with men: a narrative review. BMC Infect Dis. 2022;22(1):255. doi: 10.1186/s12879-022-07171-2 35287617 PMC8922931

[2] van Bergen JEAM, Hoenderboom BM, David S, Deug F, Heijne JCM, van Aar F, et al. Where to go to in chlamydia control? From infection control towards infectious disease control. Sex Transm Infect. 2021;97(7):501–6. doi: 10.1136/sextrans-2021-054992 34045364 PMC8543211

[3] Unemo M, Bradshaw CS, Hocking JS, de Vries HJC, Francis SC, Mabey D, et al. Sexually transmitted infections: challenges ahead. Lancet Infect Dis. 2017;17(8):e235–79. doi: 10.1016/S1473-3099(17)30310-9 28701272

[4] World Health Organization (WHO). Global progress report on HIV, viral hepatitis and sexually transmitted infections, 2021. Accountability for the global health sector strategies 2016–2021: actions for impact. Web Annex 2. Data methods. 2021.

[5] Alexiou ZW, Hoenderboom BM, Hoebe CJPA, Dukers-Muijrers NHTM, Götz HM, van der Sande MAB, et al. Reproductive tract complication risks following Chlamydia trachomatis infections: a long-term prospective cohort study from 2008 to 2022. Lancet Reg Health Eur. 2024;45:101027. doi: 10.1016/j.lanepe.2024.101027 39247903 PMC11378087

[6] Davies B, Turner KME, Frølund M, Ward H, May MT, Rasmussen S, et al. Risk of reproductive complications following chlamydia testing: a population-based retrospective cohort study in Denmark. Lancet Infect Dis. 2016;16(9):1057–64. doi: 10.1016/S1473-3099(16)30092-5 27289389

[7] Price MJ, Ades AE, Soldan K, Welton NJ, Macleod J, Simms I, et al. The natural history of Chlamydia trachomatis infection in women: a multi-parameter evidence synthesis. Health Technol Assess. 2016;20(22):1–250. doi: 10.3310/hta20220 27007215 PMC4819202

[8] Hocking JS, Temple-Smith M, Guy R, Donovan B, Braat S, Law M, et al. Population effectiveness of opportunistic chlamydia testing in primary care in Australia: a cluster-randomised controlled trial. Lancet. 2018;392(10156):1413–22. doi: 10.1016/S0140-6736(18)31816-6 30343857

[9] van den Broek IVF, van Bergen JEAM, Brouwers EEHG, Fennema JSA, Götz HM, Hoebe CJPA, et al. Effectiveness of yearly, register based screening for chlamydia in the Netherlands: controlled trial with randomised stepped wedge implementation. BMJ. 2012;345:e4316. doi: 10.1136/bmj.e4316 22767614 PMC3390168

[10] Oakeshott P, Kerry S, Aghaizu A, Atherton H, Hay S, Taylor-Robinson D, et al. Randomised controlled trial of screening for Chlamydia trachomatis to prevent pelvic inflammatory disease: the POPI (prevention of pelvic infection) trial. BMJ. 2010;340:c1642. doi: 10.1136/bmj.c1642 20378636 PMC2851939

[11] Gupta K, Wiener HW, Tiwari HK, Geisler WM. HLA-DQB1*06 and Select Neighboring HLA Variants Predict Chlamydia Reinfection Risk. Int J Mol Sci. 2023;24(21):15803. doi: 10.3390/ijms242115803 37958786 PMC10647357

[12] Morré SA. Chlamydia trachomatis complications. Int Innov Dissemin Sci Res Technol. 2015;192:46–8.

[13] Bailey RL, Natividad-Sancho A, Fowler A, Peeling RWW, Mabey DCW, Whittle HC, et al. Host genetic contribution to the cellular immune response to Chlamydia trachomatis: Heritability estimate from a Gambian twin study. Drugs Today (Barc). 2009;45 Suppl B:45–50. 20011694

[14] Morré SA, Karimi O, Ouburg S. Chlamydia trachomatis: identification of susceptibility markers for ocular and sexually transmitted infection by immunogenetics. FEMS Immunol Med Microbiol. 2009;55(2):140–53. doi: 10.1111/j.1574-695X.2009.00536.x 19170753

[15] Branković I, van Ess EF, Noz MP, Wiericx WAJ, Spaargaren J, Morré SA, et al. NOD1 in contrast to NOD2 functional polymorphism influence Chlamydia trachomatis infection and the risk of tubal factor infertility. Pathog Dis. 2015;73(1):1–9. doi: 10.1093/femspd/ftu028 25854006 PMC4542905

[16] Malogajski J, Branković I, Land JA, Thomas PPM, Morré SA, Ambrosino E. The Potential Role for Host Genetic Profiling in Screening for Chlamydia-Associated Tubal Factor Infertility (TFI)-New Perspectives. Genes (Basel). 2019;10(6):410. doi: 10.3390/genes10060410 31142036 PMC6627277

[17] Hoenderboom BM, van Oeffelen AAM, van Benthem BHB, van Bergen JEAM, Dukers-Muijrers NHTM, Götz HM, et al. The Netherlands Chlamydia cohort study (NECCST) protocol to assess the risk of late complications following Chlamydia trachomatis infection in women. BMC Infect Dis. 2017;17(1):264. doi: 10.1186/s12879-017-2376-y 28399813 PMC5387293

[18] van Ess EF, Eck-Hauer A, Land JA, Morré SA, Ouburg S. Combining individual Chlamydia trachomatis IgG antibodies MOMP, TARP, CPAF, OMP2, and HSP60 for tubal factor infertility prediction. Am J Reprod Immunol. 2019;81(3):e13091. doi: 10.1111/aji.13091 30629310 PMC6593993

[19] Anyalechi GE, Hong J, Danavall DC, Martin DL, Gwyn SE, Horner PJ, et al. High Plasmid Gene Protein 3 (Pgp3) Chlamydia trachomatis Seropositivity, Pelvic Inflammatory Disease, and Infertility Among Women, National Health and Nutrition Examination Survey, United States, 2013-2016. Clin Infect Dis. 2021;73(8):1507–16. doi: 10.1093/cid/ciab506 34050737 PMC8674123

[20] Liu C, Mokashi NV, Darville T, Sun X, O’Connell CM, Hufnagel K, et al. A Machine Learning-Based Analytic Pipeline Applied to Clinical and Serum IgG Immunoproteome Data To Predict Chlamydia trachomatis Genital Tract Ascension and Incident Infection in Women. Microbiol Spectr. 2023;11(4):e0468922. doi: 10.1128/spectrum.04689-22 37318345 PMC10434056

[21] Li P, Chen Z. Association between serum Chlamydia trachomatis antibody levels and infertility among reproductive-aged women in the U.S. Front Public Health. 2023;11:1117245. doi: 10.3389/fpubh.2023.1117245 37089503 PMC10113615

[22] Hillis SD, Owens LM, Marchbanks PA, Amsterdam LF, Mac Kenzie WR. Recurrent chlamydial infections increase the risks of hospitalization for ectopic pregnancy and pelvic inflammatory disease. Am J Obstet Gynecol. 1997;176(1 Pt 1):103–7. doi: 10.1016/s0002-9378(97)80020-8 9024098

[23] Maeda E, Miyata A, Boivin J, Nomura K, Kumazawa Y, Shirasawa H, et al. Promoting fertility awareness and preconception health using a chatbot: a randomized controlled trial. Reprod Biomed Online. 2020;41(6):1133–43. doi: 10.1016/j.rbmo.2020.09.006 33039321

[24] Harper J, Boivin J, O’Neill HC, Brian K, Dhingra J, Dugdale G, et al. The need to improve fertility awareness. Reprod Biomed Soc Online. 2017;4:18–20. doi: 10.1016/j.rbms.2017.03.002 29774262 PMC5952813

[25] Rosenstock IM. Historical Origins of the Health Belief Model. Health Educ Monogr. 1974;2(4):328–35. doi: 10.1177/109019817400200403 299611

[26] Powers JL, Tiffany JS. Engaging youth in participatory research and evaluation. J Public Health Manag Pract. 2006;Suppl:S79-87. doi: 10.1097/00124784-200611001-00015 17035908

[27] Gnoth C, Godehardt E, Frank-Herrmann P, Friol K, Tigges J, Freundl G. Definition and prevalence of subfertility and infertility. Hum Reprod. 2005;20(5):1144–7. doi: 10.1093/humrep/deh870 15802321

[28] Mayrhofer D, Holzer I, Aschauer J, Selzer C, Parry JP, Ott J. Incidence and Causes of Tubal Occlusion in Infertility: A Retrospective Cohort Study. J Clin Med. 2024;13(13):3961. doi: 10.3390/jcm13133961 38999525 PMC11242127

[29] Fishbein M, Ajzen I. Predicting and Changing Behavior. 1 ed. Ajzen I, editor. New York: Psychology Press; 2010.

[30] van Wees DA, den Daas C, Kretzschmar MEE, Heijne JCM. Who drops out and when? Predictors of non-response and loss to follow-up in a longitudinal cohort study among STI clinic visitors. PLoS One. 2019;14(6):e0218658. doi: 10.1371/journal.pone.0218658 31216341 PMC6583983

[31] Cyders MA, Littlefield AK, Coffey S, Karyadi KA. Examination of a short English version of the UPPS-P Impulsive Behavior Scale. Addict Behav. 2014;39(9):1372–6. doi: 10.1016/j.addbeh.2014.02.013 24636739 PMC4055534

[32] van Wees DA, den Daas C, Kretzschmar MEE, Heijne JCM. Double trouble: modelling the impact of low risk perception and high-risk sexual behaviour on chlamydia transmission. J R Soc Interface. 2018;15(141):20170847. doi: 10.1098/rsif.2017.0847 29618527 PMC5938578

[33] Curry I, Luk JW, Trim RS, Hopfer CJ, Hewitt JK, Stallings MC, et al. Impulsivity Dimensions and Risky Sex Behaviors in an At-Risk Young Adult Sample. Arch Sex Behav. 2018;47(2):529–36. doi: 10.1007/s10508-017-1054-x 28884246 PMC6067112

[34] Deckman T, Nathan DeWall C. Negative urgency and risky sexual behaviors: A clarification of the relationship between impulsivity and risky sexual behavior. Personal Indiv Diff. 2011;51(5):674–8. doi: 10.1016/j.paid.2011.06.004

[35] den Daas C, Häfner M, de Wit J. The impact of long-term health goals on sexual risk decisions in impulsive and reflective cognitive States. Arch Sex Behav. 2014;43(4):659–67. doi: 10.1007/s10508-013-0183-0 24081445

[36] Palinkas LA, Horwitz SM, Green CA, Wisdom JP, Duan N, Hoagwood K. Purposeful Sampling for Qualitative Data Collection and Analysis in Mixed Method Implementation Research. Adm Policy Ment Health. 2015;42(5):533–44. doi: 10.1007/s10488-013-0528-y 24193818 PMC4012002

[37] Braun V, Clarke V. Using thematic analysis in psychology. Qual Res Psychol. 2006;3(2):77–101. doi: 10.1191/1478088706qp063oa

[38] Willis GB, Artino AR. What do our respondents think we’re asking? Using cognitive interviewing to improve medical education surveys. J Grad Med Educ. 2013;5(3):353–6. doi: 10.4300/JGME-D-13-00154.1 24404294 PMC3771159

[39] van Bergen IJW, Heijne JCM, de Bruin M, van Wees DA. Impact of reduced chlamydia testing on STI testing and provider preferences in the Netherlands: an experimental vignette study. Sex Transm Infect. 2025;sextrans-2025-056703. doi: 10.1136/sextrans-2025-056703 41381200 PMC13539840

[40] Zou G. A modified poisson regression approach to prospective studies with binary data. Am J Epidemiol. 2004;159(7):702–6. doi: 10.1093/aje/kwh090 15033648

[41] Elo S, Kyngäs H. The qualitative content analysis process. J Adv Nurs. 2008;62(1):107–15. doi: 10.1111/j.1365-2648.2007.04569.x 18352969

[42] de Graaf H, Oldenhof A, Kraan Y, Beek T, Kuipers L, Vermey K. Seks onder je 25e. Uitgeverij Eburon; 2024.

[43] Darroch J, Myers L, Cassell J. Sex differences in the experience of testing positive for genital chlamydia infection: a qualitative study with implications for public health and for a national screening programme. Sex Transm Infect. 2003;79(5):372–3. doi: 10.1136/sti.79.5.372 14573831 PMC1744751

[44] Duncan B, Hart G, Scoular A, Bigrigg A. Qualitative analysis of psychosocial impact of diagnosis of Chlamydia trachomatis: implications for screening. BMJ. 2001;322(7280):195–9. doi: 10.1136/bmj.322.7280.195 11159612 PMC26583

[45] Grandahl M, Larsson M, Herrmann B. “To be on the safe side”: a qualitative study regarding users’ beliefs and experiences of internet-based self-sampling for Chlamydia trachomatis and Neisseria gonorrhoeae testing. BMJ Open. 2020;10(12):e041340. doi: 10.1136/bmjopen-2020-041340 33376171 PMC7778762

[46] Kangas I, Andersen B, Olesen F, Møller JK, Østergaard L. Psychosocial impact of Chlamydia trachomatis testing in general practice. Br J Gen Pract. 2006;56(529):587–93. 16882376 PMC1874522

[47] Bodin M, Plantin L, Schmidt L, Ziebe S, Elmerstig E. The pros and cons of fertility awareness and information: a generational, Swedish perspective. Hum Fertil (Camb). 2023;26(2):216–25. doi: 10.1080/14647273.2021.1968045 34423731

[48] Goundry ALR, Finlay ER, Llewellyn CD. Talking about links between sexually transmitted infections and infertility with college and university students from SE England, UK: a qualitative study. Reprod Health. 2013;10:47. doi: 10.1186/1742-4755-10-47 24020982 PMC3847203

[49] Lin M-H, Hwu Y-M, Lin S-Y, Lee RK-K. Treatment of infertile women with unilateral tubal occlusion by intrauterine insemination and ovarian stimulation. Taiwan J Obstet Gynecol. 2013;52(3):360–4. doi: 10.1016/j.tjog.2012.01.037 24075374

[50] Maeda E, Nakamura F, Kobayashi Y, Boivin J, Sugimori H, Murata K, et al. Effects of fertility education on knowledge, desires and anxiety among the reproductive-aged population: findings from a randomized controlled trial. Hum Reprod. 2016;31(9):2051–60. doi: 10.1093/humrep/dew133 27301362 PMC4991656

[51] Colloca L, Miller FG. The nocebo effect and its relevance for clinical practice. Psychosom Med. 2011;73(7):598–603. doi: 10.1097/PSY.0b013e3182294a50 21862825 PMC3167012

[52] Samenleving RvV. Iedereen bijna ziek Over de keerzijden van diagnose-expansie. Den Haag: RVS; 2025.

[53] Brown R. Building children and young people’s resilience: Lessons from psychology. Int J Disaster Risk Reduct. 2015;14:115–24. doi: 10.1016/j.ijdrr.2015.06.007

[54] Phillips SP, Reipas K, Zelek B. Stresses, Strengths and Resilience in Adolescents: A Qualitative Study. J Prim Prev. 2019;40(6):631–42. doi: 10.1007/s10935-019-00570-3 31659580

[55] Sharma DrLR, Bidari S, Bidari D, Neupane S, Sapkota R. Exploring the Mixed Methods Research Design: Types, Purposes, Strengths, Challenges, and Criticisms. Glob Acad J Linguist Lit. 2023;5(1):3–12. doi: 10.36348/gajll.2023.v05i01.002

[56] DeCuir-Gunby JT. Mixed methods research in the social sciences. In: Osborne JW, editor. Best practices in quantitative methods. Sage. 2008. p. 125–36.

[57] Zohrabi M. Mixed Method Research: Instruments, Validity, Reliability and Reporting Findings. TPLS. 2013;3(2). doi: 10.4304/tpls.3.2.254-262

