# Supplementary material for: Young adults’ perspectives on chlamydia-related subfertility and a potential predictive subfertility test: A mixed-methods study in the Netherlands
Source: PLoS One. 2026 Jun 18;21(6):e0351874. doi: 10.1371/journal.pone.0351874 (PMC13278409; doi:10.1371/journal.pone.0351874)
Supplement: S1 File — (DOCX) [file pone.0351874.s001.docx]

Supplementary material - Manuscript: **Young adults’ perspectives on chlamydia-related subfertility and a potential predictive subfertility test: A mixed-methods study in the Netherlands**

S1 File. Supplementary materials including: questionnaire on the predictive subfertility test (S1), topic list and focus group program (S2), PowerPoint presentation on chlamydia subfertility and the potential predictive test (S3), focus group results (S4), and table of factors associated with willingness to use the predictive test (S5).

S1. Questionnaire

| **Component** | **English question** | **English answer scale/options** | **Dutch** | **Dutch answer scale/options** |
| --- | --- | --- | --- | --- |
| **Demographics** | **What is your age?** |  | Wat is je leeftijd? |  |
| **Demographics** | **Wat is your biological sex?** | Man / Woman | Wat was je geslacht bij geboorte? (biologisch geslacht) | Man / Vrouw |
| **Demographics** | **I see myself as…** | Man / Woman / Non binary / Another gender identity, namely | Ik zie mijzelf als … | Man / Vrouw / Non-binair / Een andere genderidentiteit, namelijk … |
| **Demographics** | **Did you undergo any of the following treatments?** | Hormonal treatment / puberty blockers / breast removal or breast construction / vagina or penis reconstruction / none | Heb je een van deze behandelingen gehad? | Hormoontherapie / puberteitsremmers / borstverwijdering- of constructie / vagina- of penisconstructie / Ik heb geen van deze behandelingen gehad |
| **Demographics** | **Have you ever had sex?** | Yes / No | Heb je al eens seks gehad? | Ja / Nee |
| **Demographics** | **Who have you had sex with in the past six months?** | Men or persons with a penis / Women or persons with a vagina / Both men or persons with a penis and women or persons with a vagina | Met wie heb je seks gehad de afgelopen 6 maanden? | Mannen of personen met een penis / Vrouwen of personen met een vagina / Zowel mannen of personen met een penis als vrouwen of personen met een vagina |
| **Demographics** | **In which country or part of the world were you born?** | Netherlands / another country in Europe / Morocco / another country in Africa / Surinam / Antilles / another country in the Mid- and South Americas / Turkey / Indonesia / Japan / another country in Asia / Australia or New Zealand / I don’t know | In welk land/werelddeel ben je geboren? | Nederland / een ander land in Europa / Marokko / een ander land in Afrika / Suriname / Antillen / Een ander land in Zuid- of Midden Amerika / Turkije / Indonesie / Japan / een ander land in Azie / Australie of Nieuw Zeeland / weet ik niet |
| **Demographics** | **In which country or part of the world was your mother born?** | Netherlands / another country in Europe / Morocco / another country in Africa / Surinam / Antilles / another country in the Mid- and South Americas / Turkey / Indonesia / Japan / another country in Asia / Australia or New Zealand / I don’t know | In welk land/werelddeel is je moeder geboren? | Nederland / een ander land in Europa / Marokko / een ander land in Afrika / Suriname / Antillen / Een ander land in Zuid- of Midden Amerika / Turkije / Indonesie / Japan / een ander land in Azie / Australie of Nieuw Zeeland / weet ik niet |
| **Demographics** | **In which country or part of the world was your father born?** | Netherlands / another country in Europe / Morocco / another country in Africa / Surinam / Antilles / another country in the Mid- and South Americas / Turkey / Indonesia / Japan / another country in Asia / Australia or New Zealand / I don’t know | In welk land/werelddeel is je vader geboren? | Nederland / een ander land in Europa / Marokko / een ander land in Afrika / Suriname / Antillen / Een ander land in Zuid- of Midden Amerika / Turkije / Indonesie / Japan / een ander land in Azie / Australie of Nieuw Zeeland / weet ik niet |
| **Demographics** | **Which country, or the culture of which country, do you feel most connected to?** | Netherlands / another country in Europe / Morocco / another country in Africa / Surinam / Antilles / another country in the Mid- and South Americas / Turkey / Indonesia / Japan / another country in Asia / Australia or New Zealand / I don’t know | Met welk land, of de cultuur van welk land voel jij je het meest verbonden? | Nederland / een ander land in Europa / Marokko / een ander land in Afrika / Suriname / Antillen / Een ander land in Zuid- of Midden Amerika / Turkije / Indonesie / Japan / een ander land in Azie / Australie of Nieuw Zeeland / weet ik niet |
| **Demographics** | **What are the first two digits of your postal code?** |  | Wat zijn de eerste twee cijfers van je postcode? |  |
| **Demographics** | **What level of education are you currently enrolled in? If you are currently not enrolled in any education, please enter the level you most recently completed.** | Primary School / Secondary School / University | Welke opleiding volg je op dit moment? Als je op dit moment geen opleiding volgt, vul dan in welke opleiding je het laatst hebt afgerond. | Lagere school of basisschool / Praktijkonderwijs / VMBO / HAVO VWO Gymnasium / MBO 1 / MBO 2-4 / HBO of WO |
| **Impulsivity** | **When I am upset, I often act without thinking** | Strongly disagree – strongly agree | Als ik me ongelukkig voel, doe ik vaak dingen zonder na te denken | Helemaal mee oneens – helemaal mee eens |
| **Impulsivity** | **When I feel rejected, I will often say things that I later regret** | Strongly disagree – strongly agree | Als ik me afgewezen voel, zeg ik vaak dingen waar ik later spijt van krijg | Helemaal mee oneens – helemaal mee eens |
| **Impulsivity** | **Sometimes when I feel bad, I can’t seem to stop what I am doing even though it is making me feel worse** | Strongly disagree – strongly agree | Vaak maak ik dingen erger als ik me ongelukkig voel, omdat ik handel zonder na te denken | Helemaal mee oneens – helemaal mee eens |
| **Impulsivity** | **I blurt out things without thinking** | Strongly disagree – strongly agree | In een opwelling zeg ik vaak dingen waar ik later spijt van krijg | Helemaal mee oneens – helemaal mee eens |
| **Health goals** | **I am motivated to stay healthy** | Strongly disagree – strongly agree | Ik ben gemotiveerd om gezond te blijven | Helemaal mee oneens – helemaal mee eens |
| **Health goals** | **It is important to me to use a condom when having sex** | Strongly disagree – strongly agree | Het is voor mij belangrijk om tijdens de seks een condoom te gebruiken | Helemaal mee oneens – helemaal mee eens |
| **Health goals** | **I worry less than average about my health** | Strongly disagree – strongly agree | Ik ben minder dan gemiddeld met mijn gezondheid bezig | Helemaal mee oneens – helemaal mee eens |
| **Health goals** | **My health is important to me** | Strongly disagree – strongly agree | Mijn gezondheid is belangrijk voor me | Helemaal mee oneens – helemaal mee eens |
| **Health goals** | **I am motivated to protect myself during sex** | Strongly disagree – strongly agree | Ik ben gemotiveerd om mezelf tijdens seks te beschermen | Helemaal mee oneens – helemaal mee eens |
| **Health goals** | **It is important to me to prevent myself from contracting an STI** | Strongly disagree – strongly agree | Ik vind het belangrijk om te voorkomen dat ik een soa krijg | Helemaal mee oneens – helemaal mee eens |
| **Sexual behavior** | **What was your age when you had sex for the first time? Please give an estimation if you are unsure.** |  | Hoe oud was je toen je voor het eerst seks had? Geef een schatting als je dit niet meer precies weet. |  |
| **Sexual behavior** | **Thinking back about your sexual life until today, how often did you use condoms during sex?** | Never / Most of the time I don’t / Sometimes I do, sometimes I don’t / Usually I do / Always | Als je terugdenkt aan je seksleven tot nu toe, hoe vaak gebruikte je condooms tijdens seks? | Nooit / meestal niet / soms wel, soms niet / meestal wel / altijd |
| **Sexual behavior** | **How many different partners have you had sex with in the past 6 months?** | … partners | Met hoeveel verschillende partners heb je in de afgelopen 6 maanden seks gehad? | … partners |
| **Sexual behavior** | **With how many of these partners did you have sex for the first time in the past 6 months?** | … partners | Met hoeveel van deze partners had je in de afgelopen 6 maanden voor het eerst seks? | … partners |
| **Sexual behavior** | **How many of these partners were men?** | … partners | Hoeveel van deze partners waren mannen? | … partners |
| **Sexual behavior** | **How many times have you had sex in the past 4 weeks?** | … times | Hoe vaak heb je in de afgelopen 4 weken seks gehad? | … keer |
| **Sexual behavior** | **Think about the partner you last had sex with. Which of the following best describes you and this partner when you last had sex?** | Established partner (married, registered partnership, long relationship, relationship longer than 3 months) / New partner (relationship shorter than 3 months) / occasional partner (you are not in a relationship but you have sex more often, friends with benefits) / One-off partner (you had sex with this partner only once, one night stand) / Sex worker (someone you pay for sex) | Denk aan de partner met wie je voor het laatst seks hebt gehad. Welke van de volgende omschrijvingen past het best bij jou en deze partner toen jullie voor het laatst seks hadden? | Vaste partner (getrouwd, geregistreerd partnerschap, langdurige relatie, relatie langer dan 3 maanden) / Nieuwe vaste partner (je hebt korter dan 3 maanden een relatie) / Losse partner (een partner waar je vaker seks mee hebt maar met wie je geen relatie hebt, zoals 'friends with benefits', scharrel) / Eenmalige partner (je hebt één keer seks gehad met deze partner, one night stand) / Sekswerker |
| **Sexual behavior** | **Think about the last time you had sex. Did you and your partner use a condom?** | Yes / No / Don’t know | Denk terug aan de laatste keer dat je seks had. Hebben jij en je partner toen een condoom gebruikt? | Ja / Nee / weet ik niet meer |
| **Sexual behavior** | **Do you think your most recent partner was also having sex with someone else during the time you were having sex?** | Yes / Probably / Probably not / No / Don’t know | Denk je dat je laatste partner ook seks had met iemand anders in de periode waarin jullie seks hadden? | Ja / waarschijnlijk wel / waarschijnlijk niet / nee / weet ik niet |
| **STI (testing) history** | **Have you ever been tested for STI?** | No never / Yes, less than 3 months ago / Yes, between 3 and 6 months ago / Yes, between 6 months and 1 year ago / Yes, more than 1 year ago | Heb je ooit een soa test gedaan? | Nee / Ja, minder dan 3 maanden geleden / Ja tussen 3 en 6 maanden geleden / Ja, 6 maanden tot 1 jaar geleden / Ja, langer dan 12 maanden geleden |
| **STI (testing) history** | **Where did you get tested the last time you were tested for STI?** | At the GP / at a sexual health centre (GGD) / with a self-test that I bought myself sent to a laboratory / with a self-test that I bought myself and provided me the result directly / With a self-test that was provided to me be my the sexual health centre (GGD) / I don’t know / other … | Waar heb je de laatste keer een soa test gedaan? | Bij de huisarts / bij de soa poli van de GGD / in het ziekenhuis / met een zelfafnametest via internet die ik heb opgestuurd naar een laboratorium / met een zelfafnametest waarbij ik de uitslag direct thuis kon aflezen / weet ik niet meer / anders, namelijk … |
| **STI (testing) history** | **What was the reason for your most recent STI test?** | I had sex without a condom / I got into a new relationship / I did not want to use a condom with my partner anymore / I was pregnant / I had symptoms that matched an STI or HIV / I was afraid I had an STI or HIV / Someone who I had sex with, told be he/she had an STI or HIV / my partner wanted me to do an STI test / other … | Waarom deed je (de laatste keer) een soa-test of hiv-test? | Ik had seks zonder condoom / ik kreeg een nieuwe relatie / ik wilde geen condoom gebruiken met mijn vaste partner / ik was zwanger / ik had klachten die passen bij een soa of hiv / ik wilde zeker zijn dat ik geen soa of hiv had / ik was bang dat ik een soa of hiv had / iemand met wie ik seks had, vertelde dat die een soa of hiv had / mijn partner wilde dat ik een soa test deed / anders, namelijk … |
| **STI (testing) history** | **Wat was the reason for not doing an STI test in the past 12 months?** | I only had sex with my regular partner / I did not have sex without a condom / I did not think of it / I did not have symptoms that matched an STI or HIV / I didn’t know I could get tested / I didn’t know where to get tested / I was afraid to obtain the results of a test / I was scared to get tested / I am sure my partner does not have an STI / testing at an SHC was not available / the SHC is too far from my home / it takes too much time to get tested for an STI / I did not want to pay for an STI test | Wat was de reden dat je geen soa test hebt gedaan in de afgelopen 12 maanden? | Ik had alleen seks met mijn vaste partner / ik had geen onveilige seks / ik had daar niet aan gedacht / ik had geen lichamelijke klachten die passen bij soa of hiv / ik wist niet dat ik me kon laten testen / ik wist niet waar ik me kon laten testen / ik was bang voor de uitslag / ik vond het eng om me te laten testen / ik weet zeker dat mijn partner geen soa heeft / ik kon niet bij de GGD terecht voor een soa test / de GGD is te ver van mijn huis / het kost te veel tijd om ene soa test te doen / ik wilde niet betalen voor een soa test / anders , namelijk … |
| **Risk perception (susceptibility)** | **How likely do you think it is that you will get chlamydia in the next six months?** | No chance at all – very likely | Hoe groot is de kans dat jij in de komende 6 maanden chlamydia krijgt? | Helemaal geen kans – hele grote kans |
| **Risk perception (susceptibility)** | **Briefly explain why you think so** |  | Leg kort uit waarom je dit denkt |  |
| **Risk perception (susceptibility)** | **How likely do you think it is that you will get gonorrhea in the next six months?** | No chance at all – very likely | Hoe groot is de kans dat jij in de komende 6 maanden chlamydia krijgt? | Helemaal geen kans – hele grote kans |
| **Risk perception (susceptibility)** | **Briefly explain why you think so** |  | Leg kort uit waarom je dit denkt |  |
| **Risk perception (severity)** | **If I would get chlamydia in the next six months, it would be ….** | Mild – severe | Als ik in de aankomende zes maanden chlamydia zou krijgen, is dit voor mij … | Helemaal niet erg – heel erg |
| **Risk perception (severity)** | **Briefly explain why you think so** |  | Leg hier kort uit waarom je dit vindt |  |
| **Risk perception (severity)** | **If I would get gonorrhea in the next six months, it would be ….** | Mild – severe | Als ik in de aankomende zes maanden chlamydia zou krijgen, is dit voor mij … | Helemaal niet erg – heel erg |
| **Risk perception (severity)** | **Briefly explain why you think so** |  | Leg hier kort uit waarom je dit vindt |  |
| **Chlamydia knowlegde** | **If you don't have any (physical) symptoms, you can still have chlamydia.** | True / Not true / Don’t know | Als je geen (lichamelijke) klachten hebt, kun je toch chlamydia hebben. | Waar / Niet waar/ Weet ik niet |
| **Chlamydia knowlegde** | **Chlamydia can cause infertility in women.** | True / Not true / Don’t know | Chlamydia kan onvruchtbaarheid bij vrouwen veroorzaken. | Waar / Niet waar/ Weet ik niet |
| **Chlamydia knowlegde** | **Chlamydia will only go away after treatment with medication.** | True / Not true / Don’t know | Chlamydia gaat alleen over na behandeling met medicijnen | Waar / Niet waar/ Weet ik niet |
| **STI history** | **Have you ever had chlamydia?** | Yes, less than 3 months ago / Yes, between 3 and 6 months ago / Yes, between 6 months and 1 year ago / Yes, more than 1 year ago / No / I don’t know | Heb je zelf ooit chlamydia gehad? | Ja, minder dan 3 maanden geleden / Ja tussen 3 en 6 maanden geleden / Ja, 6 maanden tot 1 jaar geleden / Ja, langer dan 12 maanden geleden / Nee / Weet ik niet |
| **STI history** | **Have you ever had gonorrhea?** | Yes, less than 3 months ago / Yes, between 3 and 6 months ago / Yes, between 6 months and 1 year ago / Yes, more than 1 year ago / No / I don’t know | Heb je zelf ooit gonorroe gehad? | Ja, minder dan 3 maanden geleden / Ja tussen 3 en 6 maanden geleden / Ja, 6 maanden tot 1 jaar geleden / Ja, langer dan 12 maanden geleden / Nee / Weet ik niet |
| **Reproductive** | **Have you ever been pregnant?** | Yes, I have been pregnant / Yes, I am currently pregnant / No / Don’t know | Ben je ooit zwanger geweest? | Ja, ik ben ooit zwanger geweest / Ja, ik ben nu zwanger / Nee / Weet ik niet |
| **Reproductive** | **Do you wish to have children?** | Yes, at this moment / Yes, but not now / Not anymore / No / Don’t know | Heb je een kinderwens? | Ja, op dit moment / Ja, maar niet nu / Niet meer / Nee / Weet ik niet |
| **Reproductive** | **Are you subfertile, or have you ever been subfertile?** | Yes, I am less fertile / Yes, I was less fertile / No / Don’t know | Ben je verminderd vruchtbaar of ben je ooit verminderd vruchtbaar geweest? | Ja ik ben verminderd vruchtbaar / Ja ben ik ooit verminderd vruchtbaar geweest / Nee / Weet ik niet |
| **Prevention attitude** | **I think making sure that I don’t get chlamydia is…** | Very unimportant – very important | Ik vind ervoor zorgen dat ik geen chlamydia krijg.... | Heel onbelangrijk – heel belangrijk |
| **Prevention attitude** | **I think making sure that I don’t get chlamydia is…** | Very unnecessary – very necessary | Ik vind ervoor zorgen dat ik geen chlamydia krijg.... | Heel overbodig – heel noodzakelijk |
| **Prevention attitude** | **I think making sure that I don’t get chlamydia is…** | Very useless – very useful | Ik vind ervoor zorgen dat ik geen chlamydia krijg.... | Heel nutteloos – heel nuttig |
| **Prevention attitude** | **I think making sure that I don’t get chlamydia is…** | Very desirable – very undesirable | Ik vind ervoor zorgen dat ik geen chlamydia krijg.... | Heel onwenselijk – heel wenselijk |
| **Risk perception (susceptibility)** | **How likely do you think it is that you could become infertile after having a chlamydia infection?** | No chance at all – very likely | Hoe groot is jouw kans om onvruchtbaar te worden wanneer je chlamydia hebt gehad? | Helemaal geen kans – hele grote kans |
| **Risk perception (severity)** | **If I were to become infertile, it would be…** | Mild – severe | Als ik onvruchtbaar zou worden is dit voor mij… | Helemaal niet erg – heel erg |
| **Prevention attitude** | **I think preventing that I could become infertile is…** | Very unimportant – very important | Ik vind het voorkomen dat ik onvruchtbaar kan worden … | Heel onbelangrijk – heel belangrijk |
| **Prevention attitude** | **I think preventing that I could become infertile is…** | Very unnecessary – very necessary | Ik vind het voorkomen dat ik onvruchtbaar kan worden … | Heel overbodig – heel noodzakelijk |
| **Prevention attitude** | **I think preventing that I could become infertile is…** | Very useless – very useful | Ik vind het voorkomen dat ik onvruchtbaar kan worden … | Heel nutteloos – heel nuttig |
| **Prevention attitude** | **I think preventing that I could become infertile is…** | Very desirable – very undesirable | Ik vind het voorkomen dat ik onvruchtbaar kan worden … | Heel onwenselijk – heel wenselijk |
| **Prevention attitude** | **When you turn 30, you will receive an invitation to participate in the national cervical cancer screening program.**  **This can be done with a smear test at the doctor’s office or with a self-test.**  **Do you think you would like to take part in this?** | Yes / No / Don’t know yet | Als je 30 jaar bent krijg je een uitnodiging om mee te doen aan het bevolkingsonderzoek naar baarmoederhalskanker.  Dit kan via een uitstrijkje bij de huisarts of een zelftest.  Denk je dat je daaraan mee wilt doen? | Ja / Nee / Weet ik nog niet |
| **Prevention attitude** | **From your 50th birthday onwards, you will be invited to take part in the national breast cancer screening program.**  **This can be done with a scan at the hospital.**  **Do you think you would like to participate in this?** | Yes / No / Don’t know yet | Vanaf je 50e verjaardag word je uitgenodigd voor het bevolkingsonderzoek naar borstkanker.  Dit kan via een scan in het ziekenhuis. Denk je dat je daaraan mee wilt doen? | Ja / Nee / Weet ik nog niet |
| **Prevention attitude** | **I think screening programs are…** | Bad – good | Ik vind bevolkingsonderzoeken… | Slecht – goed |
| **Prevention attitude** | **I think screening programs are…** | Unnecessary – necessary | Ik vind bevolkingsonderzoeken… | Onnodig – Nodig |
| **Prevention attitude** | **I think screening programs are…** | Stressful – not stressful | Ik vind bevolkingsonderzoeken… | Stressvol – Niet stressvol |
| **Subfertility test attitude** | **Imagine yourself in the situation (Supplementary material 4).**  **If I have (had) chlamydia, I want to know if I have a higher risk of having trouble getting pregnant.** | Strongly disagree – strongly agree | Stel je de situatie voor (Supplementary material 4). Als ik chlamydia heb (gehad) wil ik weten of ik een grotere kans heb om moeilijker zwanger te worden. | Helemaal mee oneens – helemaal mee eens |
| **Subfertility test benefits** | **What would be the benefits for you of knowing your risk of having trouble getting pregnant after chlamydia?** |  | Wat zouden voor jou voordelen zijn om je kans te weten om moeilijker zwanger te worden na chlamydia? |  |
| **Subfertility test barriers** | **What could be the downsides for you of knowing your risk of having trouble getting pregnant after chlamydia?** |  | Wat zouden voor jou nadelen zijn om je kans te weten om moeilijker zwanger te worden na chlamydia? |  |
| **Subfertility test attitude** | ***Suppose the test shows that you do NOT have a higher risk of having trouble getting pregnant after chlamydia, then…***  **.. I would be less worried** | Strongly disagree – strongly agree | *Stel de test wijst uit dat je GEEN grotere kans hebt om moeilijker zwanger te worden na chlamydia, dan …*  Zou ik mijn minder zorgen maken*.* | Helemaal mee oneens – helemaal mee eens |
| **Subfertility test attitude** | **.. I would use condoms less often** | Strongly disagree – strongly agree | Zou ik minder vaak een condoom gebruiken | Helemaal mee oneens – helemaal mee eens |
| **Subfertility test attitude** | **.. I would be relieved** | Strongly disagree – strongly agree | Zou ik opgelucht zijn | Helemaal mee oneens – helemaal mee eens |
| **Subfertility test attitude** | **.. it would not change anything for me.** | Strongly disagree – strongly agree | Verandert er niks voor mij | Helemaal mee oneens – helemaal mee eens |
| **Subfertility test attitude** | ***Suppose the test shows that you DO have a higher risk of having trouble getting pregnant after chlamydia, then…***  **.. I would worry a lot** | Strongly disagree – strongly agree | *Stel de test wijst uit dat je WEL grotere kans hebt om moeilijker zwanger te worden na chlamydia, dan …*  … zou ik mij veel zorgen maken | Helemaal mee oneens – helemaal mee eens |
| **Subfertility test attitude** | **.. I would use condoms more often** | Strongly disagree – strongly agree | .. zou ik vaker een condoom gebruiken | Helemaal mee oneens – helemaal mee eens |
| **Subfertility test attitude** | **.. I could prepare better for the future.** | Strongly disagree – strongly agree | .. kan ik mij beter voorbereiden op de toekomst | Helemaal mee oneens – helemaal mee eens |
| **Subfertility test attitude** | **.. it would not change anything for me.** | Strongly disagree – strongly agree | .. verandert er voor mij niks | Helemaal mee oneens – helemaal mee eens |
| **Subfertility test attitude** | **Who do you think the test is suitable for? (multiple answers possible)** | All women with chlamydia / Women with chlamydia and a desire to have children / Women that worry about their fertility following chlamydia / Don’t know | Voor wie vind jij dat de test geschikt is? (meerdere antwoorden mogelijk) | Alle vrouwen met chlamydia / Vrouwen met chlamydia die een kinderwens hebben / Vrouwen die zich zorgen maken om hun vruchtbaarheid na chlamydia / Weet ik niet |
| **Subfertility test requirements** | **When is a good time to offer the test? (multiple answers possible)** | Shortly after women have been told they have chlamydia / When women start wanting to have children / Don’t know | Wanneer is het een goed moment om de test aan te bieden? (meerdere antwoorden mogelijk) | Kort nadat vrouwen hebben gehoord dat ze chlamydia hebben /  Wanneer vrouwen starten met een kinderwens /  Weet ik niet |
| **Subfertility test requirements** | **Reliability of the test: This is about how reliable the test is and whether it gives the correct result. For example, that you are told you do not have a higher risk, while in fact you do.**  **How reliable do you think the test should be at a minimum?** | 8 out of 10 results must be correct (80%) / 9 out of 10 results must be correct (90%) / 99 out of 100 results must be correct (99%) / Don’t know | Betrouwbaarheid van de test: Dit gaat over hoe betrouwbaar de test is en dat de test het goede resultaat geeft. Bijvoorbeeld dat je hoort dat je geen grotere kans hebt, terwijl dit wel zo is.  Hoe betrouwbaar vind jij dat de test minimaal moet zijn? | 8 van de 10 resultaten moeten kloppen (80%) /  9 van de 10 resultaten moeten kloppen (90%) /  99 van de 100 resultaten moeten kloppen (99%) /  Weet ik niet |
| **Subfertility test requirements** | **I find it unpleasant to have blood drawn for the test.** | Strongly disagree – strongly agree | Ik vind het vervelend om bloed af te laten nemen voor de test | Helemaal mee oneens – helemaal mee eens |
| **Subfertility test requirements** | **I would only take the test if it is free.** | Strongly disagree – strongly agree | Ik zou de test alleen doen als deze gratis is | Helemaal mee oneens – helemaal mee eens |
| **Subfertility test attitude** | **I think the test is a good addition to sexual health care.** | Strongly disagree – strongly agree | Ik vind de test een goede toevoeging in de zorg voor seksuele gezondheid | Helemaal mee oneens – helemaal mee eens |
| **Subfertility test intent** | **If I have (had) chlamydia, I want to take the test to know if I have a higher risk of having trouble getting pregnant.** | Strongly disagree – strongly agree | Als ik chlamydia heb (gehad) wil ik de test doen om te weten of ik een grotere kans heb om moeilijker zwanger te worden. | Helemaal mee oneens – helemaal mee eens |
| **Subfertility test intent** | **If I have (had) chlamydia and I am trying to have children, I want to take the test to know if I have a higher risk of having trouble getting pregnant.** | Strongly disagree – strongly agree | Als ik chlamydia heb (gehad) en ik bezig ben met kinderen krijgen wil ik de test doen om te weten of ik een grotere kans heb om moeilijker zwanger te worden. | Helemaal mee oneens – helemaal mee eens |

Note: A part of the questionnaire addressing demographics, sexual behavior, STI (testing) history, risk perception, health goals, and impulsivity was developed based on an existing validated questionnaire from a previous study (van Wees et al., 2019, PLOS ONE), and has also been used in a study by van Bergen I, Alexiou Z et al. (2025, *submitted*).

|  | *Time* | *Topic* | *Method* |
| --- | --- | --- | --- |
|  | 5 min | Reception, introduction, informed consent | Welcome, brief explanation of the purpose and program, introduction round |
|  | 20 min | **Prior knowlegde on STIs/chlamydia,**  **Risk perception of chlamydia (-complications)** | Individually write down answers on post-its; stick them on a flip chart; group discussion and follow-up questions by the researcher |
|  | 5 min | Explanation about chlamydia, chlamydia complications, risk of infertility, and possible future options | Presentation by researcher, using PowerPoint slides |
|  | 15 min | **Needs, wishes, and ideas about knowing your own risk**. | Group discussion |
|  | 10 min | **Barriers and benefits of a predictive test** | Describe what the advantages and disadvantages of the test could be. |
|  | 10 min | **Requirements for a predictive test** | Scenarios:  For example, suppose the test predicts correctly in 9 out of 10 cases. What do you think about that? For example, someone gets the result that they have a higher risk, but the test was wrong and that’s not actually the case.  Suppose there is no treatment or follow-up consultation—what do you think about that? |
|  |  |  |  |
|  | 10 min | **Willingness to take the test** | Open discussion and help questions   - Would you be willing to answer questions for the test? Why or why not? - Would you be willing to have blood drawn for the test? Why or why not? - Would you be willing to have a genetic test done (for example, with a cheek swab) for the test? Why or why not? |
|  | 5 min | Closing – content related | Summary of results, ask participants to add any final thoughts on advantages and disadvantages of having such a test |
| Closing | 15 min | Closing | Evaluate to learn for a next session, thank participants, provide incentive, reimburse travel expenses, explain next steps, answer any remaining questions. |

S2. Topiclist - program focus group young women

Introduction

S3. Powerpoint presentation on subfertility risk test


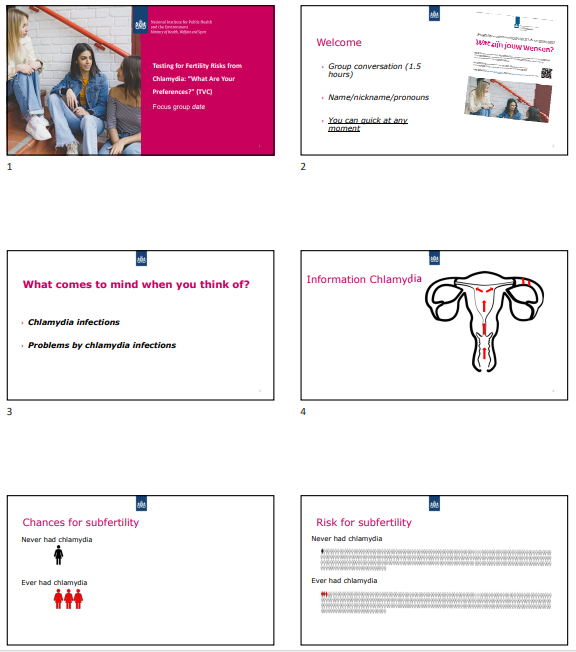


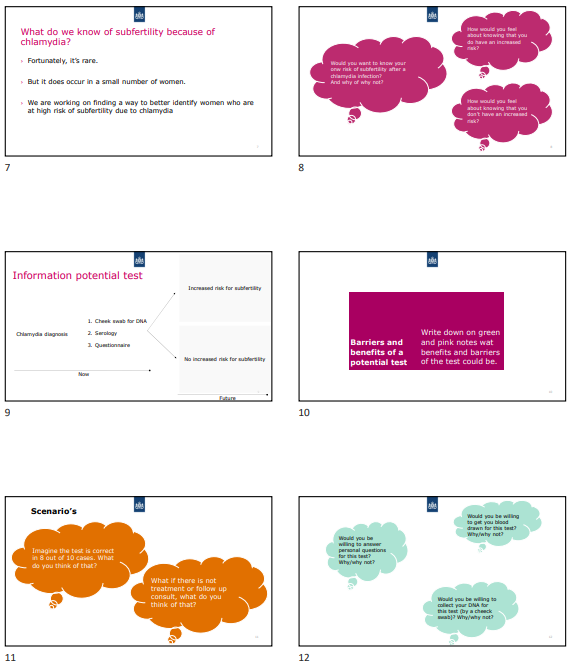


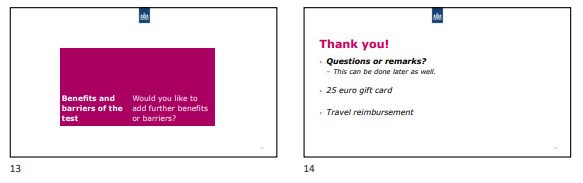


S4. Supplementary focus group results

*STI/chlamydia knowledge*

The majority of the YW were quite knowledgeable about chlamydia infections and STIs in general, although many perceived their STIs knowledge to be low. Chlamydia was believed to be very common. YW were aware of the often asymptomatic nature of the infection specifically in women, but also of possible symptoms.

*“.. And I also thought that women have fewer symptoms, so they notice it [chlamydia] less quickly, which might cause them to carry it for longer.” (FG3, P5)*

Although all YW were aware of the subfertility risk following a chlamydia infection, the actual mechanism on how chlamydia might cause subfertility was unknown. Some knew that chlamydia can resolve without treatment, while others believed to always need treatment.

*STI prevention attitudes and behaviors*

Most YW intended to protect themselves from chlamydia and other STIs. Although many YW intended to use condoms, they frequently did not. Contributing factors included being drunk during sex, have forgotten to use a condom, preferring the sensation of sex without a condom, feeling burdened towards the male partner, or having a latex allergy.

*"I think everyone wants to be good but sometimes if you have a really drunk night than you just forget it [condoms].” (FG3, P3)*

Condomless sex was not seen as negative but the possible consequences (i.e. STI/subfertility) for themselves or others were. Aside from using condoms, YW (thought they) protected themselves against (negative consequences of) STIs by getting tested regularly, assessing a partner’s physical appearance, and discussing their current STIs status.

*Perceived susceptibility chlamydia (-subfertility)*

Many women had a rather high perceived susceptibility of chlamydia and believed the overall chance of ever contracting chlamydia is about 30-50%. Reasons for this were the common nature of chlamydia, having had condomless sex, having had multiple sex partners, and the believed contagious nature of chlamydia.

*“I think there is a high chance [getting chlamydia]. I think like 50% or something. It depends of course. If you never have sex the chance is very small. But I think if you like have multiple, like if you don’t have a relationship and you have multiple partners.” FG1, R2*

Furthermore, most YW either knew peers who had contracted chlamydia, or they had experienced the infection themselves. Others were surprised to have never had chlamydia. Last, many women believed heterosexual men to care less about (preventing) STIs.

*“Yes, plus I think that for men it just doesn’t sound that serious, because they think, ‘Well, you can become infertile, but that’s just for women.’ So I don’t think they care as much. But for women, it’s actually pretty serious.” F4, P1*

In contrast to the high perceived susceptibility for a chlamydia infection, the perceived susceptibility for subfertility following chlamydia was rather low. Estimations ranged from 0.1% to 10%, often around 1%, depending on infection characteristics. Some YW believed that if you had an unknown infection for a long time, subfertility is inevitable and contracting chlamydia a second time will more significantly increases the risk of subfertility.

*“And if you get it [chlamydia] a second time than it’s a chance of 1 in 100 or something or 2 to 100 or 5. Something like that. But before I researched this I didn’t even know that chlamydia could cause infertility. [...] Maybe if you would know this in advance you can keep that into account.” FG2, R2*

*Perceived severity of chlamydia (-subfertility)*

Many women who have had chlamydia experienced negative emotions, including shame, shock from unexpected chlamydia diagnosis, or guilt over the potential transmission to others. Regardless of previous infections, the often asymptomatic nature of chlamydia created stress, fear and insecurity among YW. Some said that having symptoms might be better, because than at least you know something is wrong.

*“Stressful enough to do an STI test. Because I was thinking I might have it [chlamydia] for a long time, for years and transmit it to others and maybe experience severe complications in the future myself. While you notice nothing” FG 1, R2*

Having an asymptomatic infection in a new relationship can lead to trust issues and fears of infidelity. Informing your sex partners when you have chlamydia is believed to be the responsible thing to do. However, this can be challenging, as it may become a subject of gossip. At that point, concerns about the impact on one's reputation might outweigh worries about potential health complications.

*“Just because it’s such a taboo, I worry more about my reputation than about what it means for my body. You just don’t want people to know, even though your body is actually more important.”. F3, P3*

Conversely, because chlamydia is common, many women have friends who have had it. This makes it easier to talk about and lessens the perceived severity of having the infection themselves. For one YW, having an chlamydia infection did not bother that much at all.

However, the idea that an (asymptomatic) chlamydia infection could lead to subfertility was perceived by many as extremely concerning. Not knowing how long it takes for the infection to lead to subfertility, added to the distress. Even for several women who do not currently wish to have children, there is a strong desire to retain the option for the future, as their preferences may change. Several women expressed that they would feel angry at themselves or regretful for potentially becoming sub fertile due to a "poor decision" such as engaging in condomless sex or neglecting to get tested when they were younger.

*“Yes, exactly blame yourself for one stupid decision, well I have had more than one, but while you had one stupid decision and because of that you’re infertile or something you know. Than you can beat yourself up, I think that is a heavy burden.” FG 4, P3*

One woman expressed that subfertility could make her feel less feminine. In contrast, for some women who were certain about not wanting children, subfertility was less of a concern.

Learning more about the actual low risk of subfertility, the actual mechanism of how chlamydia might cause subfertility and possibilities for subfertility treatment options during the focus group was of reassurance and gave relief to several women. Some YW wished they knew this earlier.

*Willingness to take the chlamydia-subfertility test*

Nearly all participants wanted to know their risk for subfertility following chlamydia, despite the potential drawbacks of having this information and provided that the test was highly reliable. Many participants felt that it would be beneficial if it would be possible to improve their chances of fertility following the test result, and not just being aware of the risk. For some participants, gaining a better understanding of the actual risk of subfertility and the underlying mechanisms, during the focus group discussions, reduced their perceived need for the test.

S5. Table. Factors tested with willingness to the risk test.

|  | | Test-willingness | Crude Relative Risks | | |
| --- | --- | --- | --- | --- | --- |
|  | | n/N (%) | RR | 95%CI | P value |
| Age - tertiles | | |  |  |  |
|  | 18-21 | 156/199 (78.4) | 1 |  |  |
|  | 22-23 | 83/107 (77.6) | 0.99 | 0.87-1.12 | 0.869 |
|  | 24-25 | 94/120 (78.3) | 1.00 | 0.89-1.13 | 0.990 |
| Education | |  |  |  |  |
|  | Practical | 63/82 (76.8) | 1 |  |  |
|  | Theoretical | 270/344 (78.5) | 1.02 | 0.90-1.16 | 0.750 |
| Migrant | |  |  |  |  |
|  | Dutch | 285/361 (79.0) | 1 |  |  |
|  | Child of migrant | 33/46 (71.7) | 0.91 | 0.75-1.10 | 0.321 |
|  | migrant | 15/19 (79.0) | 1 | 0.79-1.27 | 1.00 |
| Ever chlamydia positive | |  |  |  |  |
|  | No | 229/288 (79.5) | 1 |  |  |
|  | Yes | 104/138 (75.4) | 0.95 | 0.85-1.06 | 0.348 |
| Ever gonorroea positive | |  |  |  |  |
|  | No | 313/397 (78.8) | 1 |  |  |
|  | Yes | 20/29 (69.0) | 0.87 | 0.68-1.12 | 0.294 |
| No. sex partners past six months | |  |  |  |  |
|  | 0-1 | 170/202 (84.2) | 1 |  |  |
|  | 2-3 | 89/121 (73.6) | 0.87 | 0.77-0.99 | 0.031 |
|  | > 3 | 74/103 (71.8) | 0.85 | 0.75-0.98 | 0.022 |
| Childwish | |  |  |  |  |
|  | Yes, now or later | 258/322 (80.1) | 1 |  |  |
|  | No/not anymore | 32/56 (57.1) | 0.71 | 0.56-0.90 | 0.005 |
|  | I don’t know | 43/48 (89.6) | 1.12 | 1.00-1.25 | 0.049 |
| Ever pregnant | |  |  |  |  |
|  | No | 320/406 (78.8) | 1 |  |  |
|  | Yes | 13/20 (65.0) | 0.82 | 0.60-1.14 | 0.246 |
| Subfertile | |  |  |  |  |
|  | No | 318/405 (78.5) | 1 |  |  |
|  | Yes | 15/21 (71.4) | 0.91 | 0.69-1.20 | 0.501 |
| Perc. Suscep. chlamydia | |  |  |  |  |
|  | Low | 247/307 (80.5) | 1 |  |  |
|  | Neutral | 51/73 (69.9) | 0.87 | 0.74-1.02 | 0.085 |
|  | High | 35/46 (76.1) | 0.95 | 0.80-1.12 | 0.523 |
| Perc. Sev. chlamydia | |  |  |  |  |
|  | Low | 13/19 (68.4) | 1 |  |  |
|  | Neutral | 60/86 (69.8) | 1.02 | 0.73-1.42 | 0.910 |
|  | High | 260/321 (81) | 1.18 | 0.87-1.61 | 0.287 |
| Perc. Suscep. subfertility | |  |  |  |  |
|  | Low | 68/91 (74.7) | 1 |  |  |
|  | Neutral | 185/236 (78.4) | 1.05 | 0.91-1.20 | 0.494 |
|  | High | 80/99 (80.8) | 1.08 | 0.93-1.26 | 0.318 |
| Perc. Sev. subfertility | |  |  |  |  |
|  | Low | 13/23 (56.5) | 1 |  |  |
|  | Neutral | 15/30 (50.0) | 0.88 | 0.53-1.47 | 0.636 |
|  | High | 305/373 (81.8) | 1.45 | 1.01-2.08 | 0.046 |
| Attitude prevention chlamydia | |  |  |  |  |
|  | Low | 168/225 (74.7) | 1 |  |  |
|  | High | 165/201 (82.1) | 1.10 | 0.99-1.21 | 0.063 |
| Attitude prevention subfertility | |  |  |  |  |
|  | Low | 150/217 (69.1) | 1 |  |  |
|  | High | 183/209 (87.6) | 1.27 | 1.14-1.40 | <0.001 |
| Chlamydia knowlegde | |  |  |  |  |
|  | Low | 55/73 (75.3) | 1 |  |  |
|  | Median | 236/291 (81.1) | 1.07 | 0.93-1.24 | 0.312 |
|  | High | 42/62 (67.7) | 0.90 | 0.72-1.12 | 0.336 |
| Health goals | |  |  |  |  |
|  | Lower | 122/163 (74.9) | 1 |  |  |
|  | Higher | 211/263 (80.2) | 1.07 | 0.96-1.19 | 0.205 |
| Impulsivity | |  |  |  |  |
|  | Low | 177/224 (79.0) | 1 |  |  |
|  | High | 156/202 (77.2) | 0.98 | 0.88-1.08 | 0.656 |
| Attitude population screening | |  |  |  |  |
|  | Low | 110/168 (65.5) | 1 |  |  |
|  | High | 223/258 (86.4) | 1.32 | 1.17-1.49 | <0.001 |
| No risk – less worries | |  |  |  |  |
|  | Less agreement | 41/68 (60.3) | 1 |  |  |
|  | More agreement | 292/358 (81.6) | 1.35 | 1.11-1.65 | 0.003 |
| No risk – less condom use | |  |  |  |  |
|  | Less agreement | 298/381 (78.2) | 1 |  |  |
|  | More agreement | 35/45 (77.8) | 0.99 | 0.84-1.17 | 0.947 |
| No risk – feel relieved | |  |  |  |  |
|  | Less agreement | 23/44 (52.3) | 1 |  |  |
|  | more agreement | 310/382 (81.2) | 1.55 | 1.17-2.07 | 0.003 |
| No risk – no change | |  |  |  |  |
|  | Less agreement | 275/334 (82.3) | 1 |  |  |
|  | More agreement | 58/92 (63.0) | 0.77 | 0.65-0.90 | 0.001 |
| Risk – no change | |  |  |  |  |
|  | Less agreement | 303/371 (81.7) | 1 |  |  |
|  | More agreement | 30/55 (54.6) | 0.67 | 0.52-0.85 | 0.001 |
| Risk – more condom use | |  |  |  |  |
|  | Less agreement | 114/158 (72.2) | 1 |  |  |
|  | More agreement | 219/268 (81.7) | 1.13 | 1.01-1.27 | 0.030 |
| Risk – more worries | |  |  |  |  |
|  | Less agreement | 42/74 (56.8) | 1 |  |  |
|  | More agreement | 291/352 (82.7) | 1.46 | 1.19-1.79 | <0.001 |
| Risk – better prepared | |  |  |  |  |
|  | Less agreement | 26/62 (41.9) | 1 |  |  |
|  | More agreement | 307/364 (84.3) | 2.01 | 1.50-2.71 | <0.001 |

RR = relative risks, CI = confidence intervals.
